# Aging-dependent alterations in gene expression and a mitochondrial signature of responsiveness to human influenza vaccination

**DOI:** 10.18632/aging.100720

**Published:** 2015-01-14

**Authors:** Juilee Thakar, Subhasis Mohanty, A. Phillip West, Samit R. Joshi, Ikuyo Ueda, Jean Wilson, Hailong Meng, Tamara P. Blevins, Sui Tsang, Mark Trentalange, Barbara Siconolfi, Koonam Park, Thomas M. Gill, Robert B. Belshe, Susan M. Kaech, Gerald S. Shadel, Steven H. Kleinstein, Albert C. Shaw

**Affiliations:** ^1^ Department of Pathology, Yale School of Medicine, New Haven CT 06520, USA; ^2^ Interdepartmental Program in Computational Biology and Bioinformatics, Yale School of Medicine, New Haven, CT 06520, USA; ^3^ Section of Infectious Diseases, Department of Internal Medicine, Yale School of Medicine, New Haven, CT 06520, USA; ^4^ Department of Pathology and Genetics, Yale School of Medicine, New Haven CT 06520, USA; ^5^ Center for Vaccine Development, Saint Louis University, St. Louis, MO 63104, USA; ^6^ Department of Internal Medicine, Yale School of Medicine, New Haven, CT 0652, USA; ^7^ Department of Immunobiology, Yale School of Medicine, New Haven, CT 06520, USA; ^8^ Department of Microbiology and Immunology, University of Rochester, Rochester, NY 14642, USA; ^9^ Department of Biostatistics and Computational Biology, University of Rochester, Rochester NY 14642, USA

**Keywords:** aging, frailty, influenza vaccine, gene expression, microarray, mitochondria

## Abstract

To elucidate gene expression pathways underlying age-associated impairment in influenza vaccine response, we screened young (age 21-30) and older (age ≥65) adults receiving influenza vaccine in two consecutive seasons and identified those with strong or absent response to vaccine, including a subset of older adults meeting criteria for frailty. PBMCs obtained prior to vaccination (Day 0) and at day 2 or 4, day 7 and day 28 post-vaccine were subjected to gene expression microarray analysis. We defined a response signature and also detected induction of a type I interferon response at day 2 and a plasma cell signature at day 7 post-vaccine in young responders. The response signature was dysregulated in older adults, with the plasma cell signature induced at day 2, and was never induced in frail subjects (who were all non-responders). We also identified a mitochondrial signature in young vaccine responders containing genes mediating mitochondrial biogenesis and oxidative phosphorylation that was consistent in two different vaccine seasons and verified by analyses of mitochondrial content and protein expression. These results represent the first genome-wide transcriptional profiling analysis of age-associated dynamics following influenza vaccination, and implicate changes in mitochondrial biogenesis and function as a critical factor in human vaccine responsiveness.

## INTRODUCTION

Influenza remains a major public health challenge in the 21^st^ century, with older adults at particular risk for increased morbidity and mortality. A typical influenza season results in approximately 30,000 deaths in the United States, with 90% of deaths occurring in adults over age 65 [1]. While both live attenuated and inactivated versions of the influenza vaccine are available, it is recommended that older adults receive the inactivated vaccine; unfortunately, the efficacy rates of vaccination are generally under 30%, with worsened responses in older adults who meet criteria for frailty [2, 3]. Poor vaccine efficacy in frail and non-frail older adults is related to impairments in immune responses associated with aging, termed immunosenescence. Age-associated alterations in adaptive immune responses are characterized by impaired B and T lymphopoiesis, as well as functional alterations in signaling and a marked decrease in antigen receptor gene repertoire diversity [4]. Immunosenescence also affects innate immunity, and is characterized by increased production of non-cell associated DNA, cytokines and acute phase reactants that may contribute to dysregulated innate immune activation [5]. Both adaptive and innate immunosenescence likely contribute to impaired vaccine responses and increased morbidity and mortality from infectious diseases among older adults. However, the molecular pathways underlying impaired vaccine responses among older adults remain incompletely understood. Elucidation of these pathways would identify potential targets for interventions designed to improve immune responses in older adults.

Systems vaccinology approaches have begun to identify gene signatures that correlate with hemagglutination-inhibition (HAI) antibody titers or viral neutralization assays post-vaccination [6-8]. Some signatures are common to different vaccines, while others are specific to influenza vaccination [9]. A signature for type I interferons early after vaccination, and a plasma cell signature seven days post-vaccination, have been observed following influenza vaccination in multiple studies [6, 7, 9]. While most of these studies have focused on young adults, a recent study including older subjects focused on the predictive power of pre-vaccination pathway activity [10]. Here, we have employed transcriptional profiling analyses in young (age 21-30) and older (age ≥ 65) adults using blood samples drawn prior to and at multiple time-points following influenza vaccine administration to provide, to our knowledge, the first genome-wide temporal assessment of vaccine response in the context of aging.

## RESULTS

### Age is a strong determinant of vaccine response

**Figure 1:**
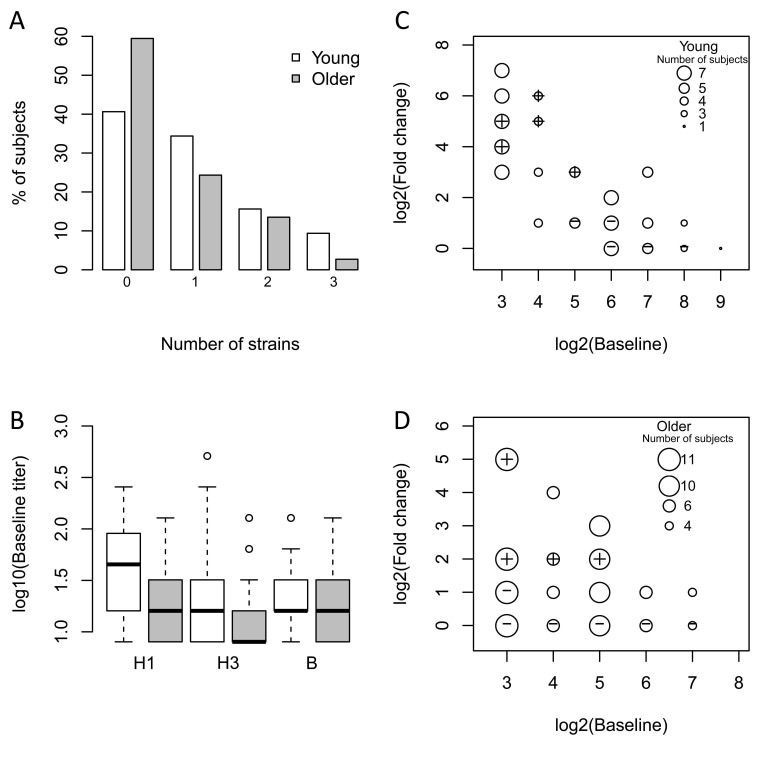
Older subjects have lower baseline titers and a reduced vaccine response The influenza antibody response to all three vaccine strains in the seasonal vaccine was measured pre-vaccination and 28 days post-vaccination by HAI. (**A**) The percentage of young (white bars) and older (grey bars) subjects showing ≥4-fold increase in HAI assay on day 28 to the indicated number of strains. (**B**) Baseline titers for each of the three strains among young (white bars) and older (grey bars) subjects. Medians are indicated by the bold line, while the box boundaries indicate the 25^th^ (lower line) and 75^th^ (upper line) percentiles, with outliers indicated by (o). (**C**) and (**D**) Comparison of baseline (pre-vaccination) HAI titers with fold-change (day 28 vs. pre-vaccination). The size of the circles (small to large) depicts the number of subjects (1 to 7 for young and 4 to 11 for older). The set of vaccine responders and non-responders selected for microarray experiments are indicated by the plus and cross within the circle symbols, respectively. For simplicity, the response against the strain that induced the maximum fold-increase in HAI titer on day 28 in vaccine responders is plotted. For non-responders, the highest baseline titer among the three vaccine strains is depicted.

We recruited 121 young (21-30 years old, n = 59) and older (≥ 70 years old, n = 62) subjects in two consecutive vaccination seasons (n = 49 in 2010-2011; n = 72 in 2011-12) prior to immunization with the seasonal trivalent inactivated influenza vaccine (TIV). Older subjects were further classified for the geriatric syndrome of frailty using the clinically validated, operational definition of Fried et al. [11]. To assess the response to vaccination, antibody titers to the three viral strains in the vaccine (A/California/7/09 (H1N1)-like virus; A/Perth /16/2009 (H3N2); and B/Brisbane/60/2008), which were the same for both seasons, were measured pre-vaccination and 28 days post-vaccination by hemagglutination inhibition (HAI). In both seasons, pre-vaccination anti-H1 titers in older subjects were significantly lower than in young subjects (2010-11: p=0.015, 2011-12: p=0.002), while titers against the H3 and B strains were similar in both age groups (Figure 1B). After vaccination, 41% of young subjects and 59% of older subjects failed to show a four-fold increase in post-vaccine HAI titer to any of the three strains in the vaccine (Figure 1A). Among these non-responders, 92% of young and 36% of older subjects had pre-existing antibody titers greater than 1:16 against at least one of the three vaccine strains (Figures 1C and 1D). Thus, while a similar frequency of young and older subjects failed to show increases in antibody titers following vaccination, most of the young subjects had elevated pre-vaccine antibody titers against the vaccine strains.

To define the transcriptional program underlying a successful influenza vaccination, and how this might be altered with age, we identified a subset of individuals with particularly strong vaccine responses and a subset with poor responses for genome-wide transcriptional profiling. Strong vaccine responders were defined as having a four-fold or greater increase in HAI (comparing titers at day 28 to day 0) for at least two of three influenza vaccine strains, while vaccine non-responders were defined as having a less than four-fold increase in HAI titers for all three vaccine strains following immunization. Using these stringent criteria, we identified 10 responders and 6 non-responders for transcriptional profiling in the 2010-2011 cohort. In the 2011-12 cohort, transcriptional profiling was carried out on 11 responders and 21 non-responders; clinical characteristics of these individuals are outlined in Table 1. Peripheral blood mononuclear cells (PBMC) were obtained pre-vaccination (day 0) and at days 2 (2011-12 cohort) or 4 (2010-11 cohort), 7 and 28 post-vaccination for gene expression microarray analysis. We focused our initial analysis on the 2011-12 season, as this cohort contained subjects with a range of vaccine responses in both young and older subjects. Specifically, we segmented the cohort into five groups: young responders (n=5), young non-responders (n=7), older responders (n=6), older non-responders (n=10) and frail non-responders (n=4). Because the smaller 2010-11 cohort contained predominantly young responders (n = 8) and older non-responders (n=5), it was used as an independent cohort to validate hypotheses generated in the 2011-12 cohort.

**Table 1:** Characteristics of the subset of 32 young and older adults in the test cohort selected from 72 participants for gene expression microarray analysis using a stringent definition of vaccine response. Note that four participants in the older group (all vaccine non-responders) met criteria for frailty

Patient Characteristics(N=32)	Young(N=12)	Older(N=20)	Probability^a^
Age (years) range (22-93), mean (SD)	25.75 (2.56)	75.10 (7.09)	<0.0001
Female, n (%)	6 (50.00)	6 (50.00)	1.000
Non-white Race, n (%)	4 (33.33)	4 (20.00)	0.4325
Influenza Vaccine Responder^b^, n (%)	5 (41.67)	6 (30.00)	0.7026
Number of Comorbid Conditions, mean (SD)	0.58 (0.90)	3.85 (1.98)	<0.0001
Number of Prescription Medications, mean (SD)	0.50 (0.67)	4.50 (2.04)	<0.0001
Number of OTC Medications, mean (SD)	0.67 (0.78)	3.65 (1.76)	<0.0001
Aspirin, n (%)	0 (0.00)	14 (70.00)	0.0003
Statin Medications, n (%)	0 (0.00)	13 (65.00)	0.0011

a Probability for Student t-test (Continuous Measures), Chi-square test/Fisher's Exact test (Categorical Measures)

b A vaccine responder had a four-fold increase in hemagglutination inhibition (HAI) titer to at least two of the three strains in the seasonal vaccine. The remaining participants analyzed were all nonresponders without a four-fold increase in HAI titer to any strain in the vaccine.

**Figure 2:**
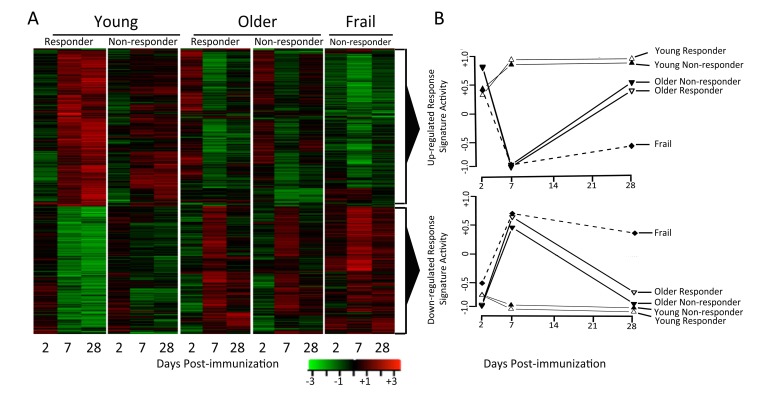
Young and older subjects exhibit qualitatively different responses to influenza vaccination Differentially-expressed genes were determined by comparing gene expression levels pre- and post-vaccination (∣fold-change∣ > 1.25 and FDR<0.05). Row-normalized fold-changes for all differentially expressed genes (rows) at each time point (columns) in the indicated cohorts are indicated in the heatmaps. Up-regulated or down-regulated response signatures were defined as the set of genes significantly up- or down-regulated in young responders. GSEA [35] was used to calculate the enrichment of these signatures in young, older and frail adults, plotted for responder and non-responder status to vaccine in the two graphs in the right-most column, where zero activity of up-regulated or down-regulated response signatures denotes absence of enrichment (note that all frail individuals were vaccine non-responders).

Differentially-expressed genes (Supplementary Table 1) were identified in each of the five response groups by comparing expression levels at each time-point post-vaccination to their pre-vaccination levels using criteria described in Methods. Overall, we identified 2,542 genes that were differentially expressed following vaccination in young subjects, and 321 differentially expressed genes in older subjects (Figure 2A). Thus, young subjects exhibited a more robust gene expression program in response to influenza vaccination.

### The temporal gene expression response to vaccination is altered with age

**Figure 3:**
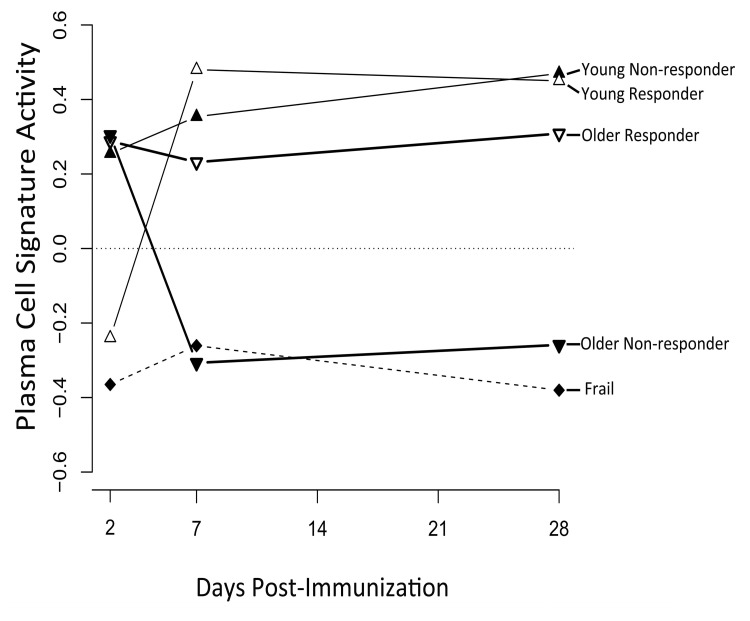
A plasma cell gene signature is enriched among vaccine responders GSEA [35] was used to calculate the enrichment of the plasma cell signature from Nakaya et al. [7] in young, older and frail cohorts, broken down by responder and non-responder status. A signature activity of zero indicates the absence of enrichment. False discovery rates by GSEA were statistically significant for young vaccine responders at day 7 (FDR=0.01) and day 28 (FDR=0.03) post-vaccine, for young non-responders at day 28 (FDR=0.02) post-vaccine, and for frail non-responders at day 28 (FDR=0.04) post-vaccine. FDR values for all other time points and groups were not significant (FDR > 0.05).

We next sought to investigate the temporal dynamics of a successful vaccination response, and how this was altered by age. Two clusters of genes were found by hierarchical clustering of differentially expressed genes across different age groups and response groups (Figure 2). The first cluster or “response signature” consisted of 810 genes that were significantly induced at day 7 post-vaccination in young responders. Some of these gene expression changes likely reflected the transient migration of plasmablasts in the blood, which occurs between days 6-11 post-vaccination [12]. Indeed, plasma cell signature genes [7] were significantly enriched at day 7 post-vaccination in young responders (Figure 3). Surprisingly, 616 of the genes that were induced at day 7 remained significantly up-regulated at day 28 post-vaccination.

The response signature displayed a qualitatively different pattern of activity in older subjects, with significant activity observed at day 2 post-vaccination (q<0.05), followed by a trend toward down-regulation on day 7 and then up-regulation on day 28 (Figure 2). Interestingly, the response signature was never significantly induced at any time-point in frail individuals (Figure 2). These patterns in older adults were independent of vaccine response status, and were confirmed for young responders and older non-responders in the 2010-11 cohort (data not shown).

The second cluster was slightly up-regulated on day 2 in young subjects and was down-regulated on day 7 and 28. Notably, in older and frail adults, up-regulation of this cluster was delayed until day 7. Pathway analysis of this cluster revealed activation of several immune pathways such as NOD-like receptor signaling and T cell and B cell receptor signaling.

To test whether age-dependent differences in the transcriptional program induced by vaccination were associated with altered gene expression levels pre-vaccination, we directly compared baseline gene expression levels in young versus older adults and identified 40 differentially expressed genes (Supplementary Table 2). Interestingly, 31 genes up-regulated in older, compared to young subjects and only one up-regulated (LRRN3) in young relative to older subjects at baseline were also up-regulated in the response signature, suggesting that some vaccine-induced genes are already elevated at baseline in older adults. Notably, the NOD-like receptor and Toll-like receptor pathways showed higher basal expression in older adults in both the 2010-11 and 2011-12 cohorts. No pathways were differentially expressed between older and frail adults.

### Pathways involved in age-specific vaccination response

**Figure 4:**
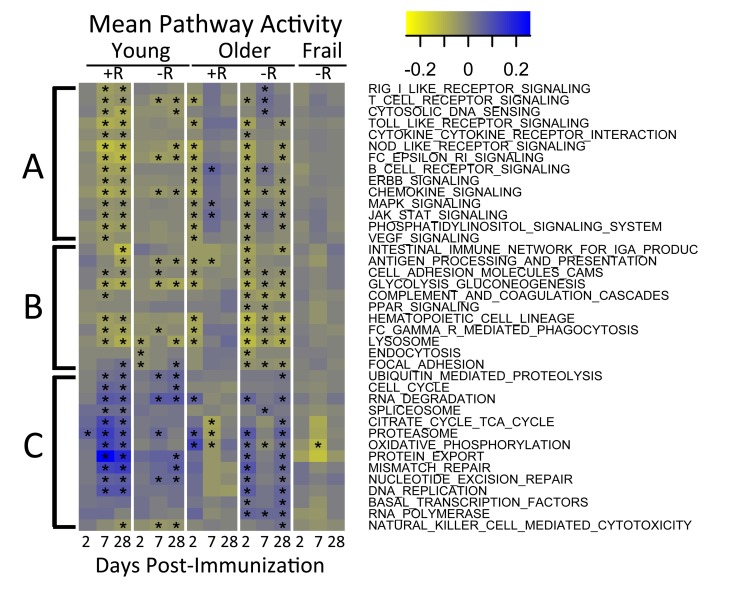
Pathway activities in response to vaccine QuSAGE [34] was used to screen a subset of immunological pathways from the KEGG database to identify pathways with significantly (FDR<0.005) altered activity (rows) during one or more time-points (x-axis) in at least one of the age-groups. Three major clusters indicated by **A**, **B** and **C** were detected. Coloring represents down-regulated (yellow) to up-regulated (blue) activity relative to the pre-vaccination time-point. +R indicates vaccine responder status, -R indicates non-responder status. See Supplementary Table 4A for a tabulation of pathway activities and Supplementary Table 4B for a list of associated FDR values. In the figure, time-points with FDR<0.05 are indicated with an asterisk.

To identify molecular mechanisms underlying vaccination response in young and older adults, we evaluated the temporal activity of blood transcription modules (BTMs, [13]) and immune-related pathways. We first evaluated a type I interferon response module (M127), in view of previous findings suggesting early upregulation of interferon stimulated genes following influenza vaccination. We found this module was significantly induced at day 2 post-vaccine specifically in young responders (p=0.01). Further BTM analyses revealed induction of B and T cell-associated modules, beginning at day 7 post-vaccine, in young vaccine responders that was not found in young non-responders. Interestingly, no significant induction of T cell module activity was found in older vaccine responders, and B cell module activity was induced at day 2 instead of day 7; in addition, modules related to monocyte and myeloid cell activity were induced at day 28 (e.g. M4.3, M11.0, and S4; Supplementary Figure 1 and Supplementary Tables 3A and 3B). Analysis of pathway activity showed one cluster (cluster A) whose temporal patterns followed the down-regulated response signature (compare Figures 2 and 4). Cluster A included “MAPK signaling” and “JAK-STAT signaling”, as well as innate immune pathways such as TLR-signaling, and had higher activities at day 7 in older responders. Cluster B, including “Fc gamma R mediated phagocytosis” and lysosome-associated pathways, appeared similar to cluster A but showed lower activity, particularly in older non-responders compared to older responders. Cluster C contained several metabolic pathways and showed a temporal pattern that followed the up-regulated response signature, with higher activity at day 7 in young adults and day 2 in older adults (Figure 4 and Supplementary Tables 4A and 4B). The pathways in cluster C, including “TCA cycle” and “Oxidative phosphory-lation”, were strongly induced in young vaccine responders but not in young non-responders. However, non-responders showed a qualitatively similar dynamic pattern of pathway activity compared to age-matched responders.

### Increased mitochondrial biogenesis in responders

**Figure 5:**
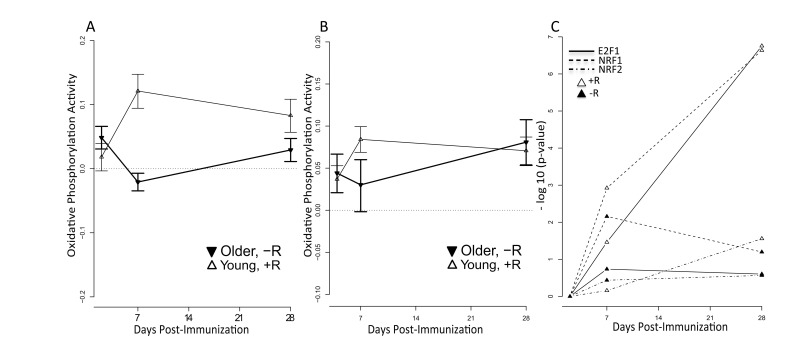
Increased mitochondrial activity among young vaccine responders QuSage [34] was used to quantify activity of the KEGG oxidative phosphorylation pathway for young responders (open symbols) and older non-responders (filled symbols). +R indicates responder status and –R non-responder status. Results are shown for (**A**) the 2011-12 cohort and (**B**) the independent 2010-11 cohort. FDR values for (**A**) are provided in Supplementary Table 4B. For the 2010-11 cohort (**B**), statistically significant p values in young responders were at day 2 (p = 1.1e-05), day 7 (p = 2.3e-11), and day 28 (p = 2.4e-09) post-vaccine. For older non-responders significant p values were at day 2 (p=0.005) and day 28 (p = 2.1e-05) post-vaccine; the p value at day 7 was not significant (p > 0.05). (**C**) Over-representation (−log_10_(p-value)) of NRF1 (dashed lines), NRF2 (dash-dot lines) and E2F1 (solid lines) target genes calculated by the hypergeometric test in responders (open symbols) and non-responders (filled symbols) across time (x-axis). Target gene sets for NRF1, NRF2 and E2F1 were defined by the presence of promoter-region binding sites defined by V$NRF1_Q6, V$NRF2_Q4 and V$E2F1_Q4_01 TRANSFAC matrices, respectively. Significant p values were found for E2F1 in young responders at day 7 (FDR = 0.03) and day 28 (FDR = 1.76e-07) post-vaccine; for NRF1 in young responders at day 7 (FDR = 0.001) and day 28 (FDR = 2.3e-07) post-vaccine and in young non-responders at day 7 (FDR = 0.007) post-vaccine; and for NRF2 in young responders at day 28 (FDR = 0.03) post-vaccine. All remaining p values did not achieve statistical significance.

We found that the oxidative phosphorylation (OXPHOS) pathway was significantly induced in vaccine responders in both the 2009-10 and 2010-11 cohorts (Figure 5A and 5B). To further explore the relevance of this pathway to a successful vaccination response, we assessed the activity for the nuclear respiratory factor 1 (NRF1), nuclear respiratory factor 2 (NRF2) and E2F1 transcription factors by examining the behavior of their target genes. NRF1 and NRF2 function as transcription factors that activate the expression of key nuclear-encoded genes involved in cellular respiration, heme biosynthesis, and mitochondrial DNA (mtDNA) transcription and replication. We observed significantly higher enrichment of target genes of all three transcription factors in responders compared to non-responders (p<0.05) in young subjects, implicating the activation of these factors in successful vaccination responses (Figure 5C). We also observed significant enrichment of target genes for other nuclear factors involved in regulation of mitochondrial biogenesis, such as CREB and YY1 in responders. However, induction of NRF1 appeared to be PGC1-alpha independent, since target genes of PGC1-alpha were not enriched in young responders or non-responders. These results support the importance of the OXPHOS signature in responding individuals.

**Figure 6:**
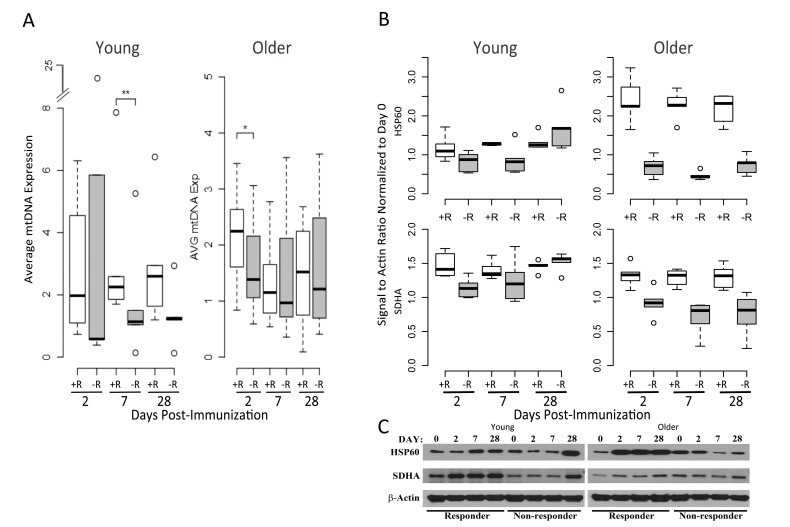
Mitochondrial DNA and OXPHOS protein abundance confirm the elevated OXPHOS signature of responders (**A**) Relative mtDNA abundance in young (left panel) and older subjects (right panel) is significantly altered between vaccine responders (open boxes) and non-responders (filled boxes) at day 7 for young responders, with a trend at day 2 for older responders, compared to non-responders. mtDNA values are normalized with respect to the pre-vaccination time point. A subset from the cohort of older responders (n=7), older non-responders (n=8), young responders (n=6) and young non-responders (n=5) was evaluated. Dashed lines indicate the lower and upper range of values, with outliers indicated by open circles, bars indicating the 25^th^ and 75^th^ percentiles and dark horizontal lines the medians ** p<0.05, *p<0.1 (**B**) Induction of the mitochondrial chaperone protein HSP60 and mitochondrial Complex II component SDHA after influenza vaccination in young (left panel) compared to older adults (right panel), stratified by vaccine status (n=5 for each of the categories of older responder, older non-responder, young responder and young non-responder; relative pixel intensities of HSP60 and SDHA using β-actin as a loading control are normalized to day 0). For HSP60, a statistically significant increase in protein expression was found for young responders compared to non-responders at day 7 (p=0.04), and for older responders compared to non-responders at days 2 (p=0.007), 7 (p=0.0001), and 28 (p=0.0001). For SDHA, a significant increase in expression in young responders compared to non-responders was found at day 2 (p=0.007), and in older responders vs. older non-responders at days 2 (p=0.006), 7 (p=0.002), and 28 (p=0.007; p values were calculated using a t-test). (C) Representative Western blot of HSP60 and SDHA expression for young and older vaccine responders and non-responders.

To experimentally validate the OXPHOS gene expression signature of the responders and determine if this was associated with increased mitochondrial biogenesis (i.e. more mitochondria/cell), we examined mitochondrial DNA (mtDNA), which encodes 13 essential proteins of the OXPHOS chain and exists in multiple copies that usually correlate with organelle amount [14, 15]. Real-time qPCR analysis of mtDNA content in a subset of our cohort showed a significant increase in mtDNA copy number of young responders compared with young non-responders at 7 days post-vaccination (Figure 6A). In addition, older responders displayed elevated mtDNA copy number at day 2 post-vaccination, correlating with induction of the OXPHOS gene signature in this group (Figure 6A). We subsequently found that expression of HSP60, a mitochondrial matrix chaperone, was significantly elevated in protein extracts analyzed in a subset of responder PBMCs, compared to non-responders (at day 7 post-vaccination in young subjects and on all days in older subjects). In addition, mitochondrial succinate dehydrogenase subunit A (SDHA), a subunit of mitochondrial OXPHOS complex II, was also significantly elevated at the protein level in young responders vs. young non-responders at day 2 after vaccination, as well as in older responders vs. older non-responders at day 2, 7 and 28 (Figure 6B and 6C). This correlated with SDHA and many other OXPHOS genes being elevated based on transcriptional profiling. Taken together, these data are consistent with a role for mitochondrial biogenesis, perhaps driving increased TCA cycle and OXPHOS activity, in vaccine responders.

### Age-independent, response-specific alterations in metabolic gene-expression

**Figure 7:**
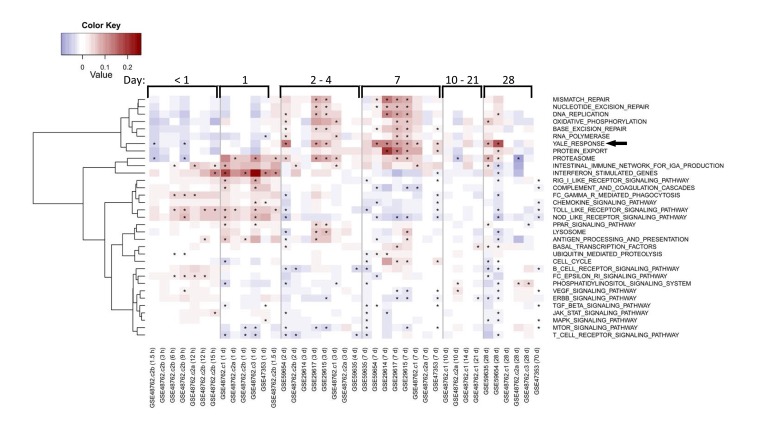
Enrichment of response and OXPHOS signature in published studies of influenza vaccine response Each row indicates a pathway gene set and each column represents a separate publicly available dataset (indicated by GEO accession number) evaluating influenza vaccine response. Average fold-changes for a gene set with respect to the study-specific baseline are shown for varying times after vaccination as indicated for general ranges at the top of the figure and in hours (h) or days (d) at the bottom of the figure following each GEO accession number. QuSAGE [34] was used to assess the activity of a subset of immunological pathways from the KEGG database and response signature in published studies, with significance (FDR<0.05) depicted by an asterisk. Pathways enriched in a minimum of five columns are depicted. The vaccine response signature described in this report is denoted as “Yale Response” (arrow). Coloring represents down-regulated (blue) to up-regulated (red) activity relative to the pre-vaccination time-point.

Our pathway analysis predicted that successful vaccination responses would be associated with early activation of antiviral pathways followed by activation of metabolic pathways. We sought to confirm these predictions using previously published gene expression data from human studies of influenza vaccination [7-9, 16]. This analysis confirmed the induction of genes in our identified response signature at day 7 (Figure 7). In some cohorts, these genes were induced as early as day 2 post-vaccination, but returned to baseline levels in most cohorts by day 28. Many antiviral pathways were activated starting around 1 day post-vaccination, and returned to baseline levels by day 2 post-vaccination. Notably, the “Oxidative phosphorylation (OXPHOS)” signature we found to be induced on day 7 in young responders was also enriched in other datasets examined (Figure 7).

## DISCUSSION

We have carried out the first systems-level assessment of alterations in gene expression prior to and following administration of the seasonal trivalent inactivated influenza vaccine including both young (age 21-30) and older (age ≥ 70) adults. We screened 121 participants to identify 51 young and older subjects who met a stringent definition of vaccine response (at least a four-fold increase in post- versus pre-vaccine HAI titer for at least two of three strains in the seasonal vaccine), or non-response (no four-fold increase in post-vaccine titer to any of the three vaccine strains). Consistent with previous observations, we found that older subjects are frequently non-responders to vaccine. Interestingly, young non-responders had higher baseline titers to at least one of the vaccine strains (frequently the H1N1 strain), suggesting in some cases that non-response could reflect inability to further increase an already elevated baseline HAI titer. We tried to minimize this potential effect through our selection of responders and non-responders. For example, the highest pre-vaccine titer found in a young non-responder analyzed in our cohort was 1:256 for one vaccine strain (Figure 1); however, the two other strains showed four- to eight-fold lower baseline titers and a lack of four-fold response. Similarly, the highest baseline titer found in an older non-responder was 1:128 for one strain, but the two other strains showed no change following vaccination with baseline levels eight- to sixteen-fold lower. Thus, using these stringent definitions of vaccine response and non-response, we sought to elucidate gene expression signatures associated with successful vaccine antibody response, and whether such signatures are affected by age.

We identified two clusters of genes, one induced on day 2 and the other on day 7 in young individuals—particularly in vaccine responders—which were enriched for innate and adaptive immune pathways (Figure 4 and Supplementary Tables 4A and 4B). Notably, the day 7 signature was induced in older responders at day 2 with non-significant trends toward down-regulation at day 7 and up-regulation at day 28, suggesting age-associated dysregulation (Figure 4). No vaccine responders were found for subjects meeting criteria for the geriatric syndrome of frailty, characterized by impaired stress resistance and increased mortality and disability, and older frail non-responders to vaccine showed a complete absence of induction of this signature. These findings extend previous work identifying pre-vaccine gene expression patterns associated with influenza vaccine response in older adults [10] by analyzing samples obtained at both pre-and post-vaccine time points to provide a picture of immune system activation by the trivalent inactivated influenza vaccine. It is conceivable that previous exposures to influenza, expected to be more extensive in older adults, could contribute to some alterations in influenza vaccine response; for example, the composition of the vaccine used in both the 2010-11 and 2011-12 cohorts contained the A/California/7/09 (H1N1) strain for which older adults have been reported to have increased titers of cross-reactive antibodies [17, 18]. However, in our cohort older adults generally had decreased pre-vaccine HAI titers compared to young adults and, for the transcriptional profiling, we specifically selected older individuals who failed to mount a fourfold increase (and in the majority of cases, had no change) in HAI titer to all strains in the vaccine, including the A/California/7/09 strain. Combined with evidence for dysregulated innate immune responses in older adults (also less likely to reflect previous exposure to influenza), we believe our results indicate that many molecular mechanisms underlying vaccine response differ between young and older adults.

Our analyses revealed elevation of an type I interferon response at day 2 post-vaccine and a plasma cell signature at day 7, in young vaccine responders—findings in agreement with previous studies of influenza vaccine response in young cohorts [6-8, 19]. BTM analysis revealed activation of modules associated with B and T cells, particularly beginning at day 7 in young responders. This pattern was altered in older responders, with induction of B cell modules at day 2, monocyte and myeloid modules at day 28, and a lack of T cell module induction. Interestingly, we also observed a novel age-independent response specific signature enriched in oxidative phosphorylation (OXPHOS) genes. This OXPHOS signature was enriched at day 7 and 2 post-vaccine in young and older responders, respectively, compared to non-responders and was validated in two independent cohorts from two successive influenza vaccine seasons in which the composition of the vaccine was unchanged. In addition, targets of the NRF1 and NRF2 transcription factors, governing OXPHOS activity via activation of nuclear genes involved in mitochondrial biogenesis and metabolism, were significantly enriched in young responders [20]. Moreover, several other nuclear factors involved in mitochondrial biogenesis such as YY1, CREB and E2F1 were also enriched in responders. That the OXPHOS signature appears to be driven by an increase in mitochondrial biogenesis is indicated by a significant increase in relative mtDNA content in PBMCs from young vaccine responders, compared to non-responders, at day 7 post-vaccine. In older responders versus non-responders, an increase in mtDNA (normalized to day 0 content) was found at day 2 but not day 7–potentially reflecting a differential OXPHOS response, though this day 2 increase did not reach statistical significance. The substantial increase in both SDHA and Hsp60 protein expression at days 2 and 7 post-vaccine, relative to day 0, in both young and older vaccine responders is also consistent with an overall increase in mitochondrial biogenesis. However, this increase was blunted or absent in non-responders, particularly in the older group (Figure 6). Taken together, these findings indicate a novel role for enhanced mitochondrial biogenesis in the response to influenza vaccination in humans. Whether this indicates an enhanced need for TCA cycle and OXPHOS activity or some other critical metabolic or signaling function (e.g. innate immune signaling, discussed below) that mitochondria provide remains to be determined. In this regard, it is noteworthy that the mitochondrial biogenesis response is PGC-1α-independent. There are other PGC-1-related proteins with non-overlapping functions [20]. For example, PRC also co-activates NRF1 and NRF2 and has been implicated in pro-inflammatory responses [21]; it is attractive to speculate that this could contribute to the unique mitochondrial biogenesis response observed in this study.

The identity of the cell type(s) responsible for the OXPHOS signature remains to be determined. In this regard, it is notable that metabolic differences have been reported in T cell subsets, with a predominance of oxidative phosphorylation associated with resting or memory T cells and aerobic glycolysis associated with activation and T helper subsets [22]. Thus, the OXPHOS signature could reflect metabolic priming in memory T cells; however, activation of another T or B cell subset(s) remains possible. This signature could also indicate a need to alter mitochondrial biogenesis or dynamics to properly activate innate immune responses, as mitochondria are critically involved in innate immune signaling [23]. Nonetheless, the OXPHOS signature could serve as a biomarker for a successful vaccine response, and raises the possibility that interventions targeting mitochondrial pathways could enhance vaccine efficacy in older adults. One potential example of such an agent would be bezafibrate, a compound used to treat hyperlipidemia that stimulates mitochondrial biogenesis through its activity as a PPAR agonist [24]. In addition, mitochondria-derived reactive oxygen species (mROS) are required for normal physiology and cellular signaling [25], and could be modulated during aging. Several lines of evidence point to a role of mROS in proliferation and production of IL-2 following TCR/CD28 stimulation, indicating that mROS are required during early stages of T cell activation. Additionally, oral administration of antioxidants in mice reduced the expansion of T cells following viral infection, suggesting that ROS are required for T cell function in vivo [26]. These results, coupled with our findings, suggest that the enhanced expression of OXPHOS genes upon vaccination may be required to rewire mitochondrial metabolism to fine-tune mROS levels, thus properly licensing antigen receptor signaling and activation of T and B cells. Integrating these transcriptional profiling data with genotyping information could provide further insights [27]. Additional experiments should also clarify whether alternate formulations of influenza vaccine developed for older adults, such as high-dose or intradermal preparations [28], are able to modify these alterations in gene expression.

## MATERIALS AND METHODS

### 

#### Clinical Study Design and Recruitment of Participants

Healthy adults (older ≥ 70 years of age, young 21-30 years) were recruited at seasonal influenza vaccination clinics organized by Yale University Health Services during the 2010 – 2011 and 2011-2012 vaccine seasons. Participants with an acute illness two weeks prior to recruitment were excluded from the study, as were individuals with primary or acquired immune-deficiency, use of immunomodulating medications including steroids or chemotherapy, a history of malignancy other than localized skin or prostate cancer, or a history of cirrhosis or renal failure requiring hemodialysis. All older subjects enrolled in the study were evaluated for frailty using the Fried protocol [11]. Briefly, frailty was determined based on a 5-point scale ranging from 0 to 5, with 1 point for each of five frailty criteria, including exhaustion (with or without effort), decreased grip strength, slow gait speed, report of decreased physical activity, and self-reported involuntary weight loss (>10lb in past one year)—all assessed using standardized instruments. Participants with zero criteria were considered non-frail, while those with 3 or more criteria were considered frail.

Blood samples were collected into Vacutainer sodium heparin tubes and serum tubes (Becton Dickinson) at four different time points, immediately prior to administration of vaccine (day 0) and on days 2 (2011-2012), 4 (2010-2011) 7 and 28.

#### Isolation of Biological samples

##### Peripheral Blood Mononuclear Cells (PBMC)

Peripheral Blood Mononuclear Cells (PBMCs) were isolated from heparinized blood using Histopaque 1077 (Sigma) gradient centrifugation as described earlier [29-31]. PBMCs were washed once with 1x Hank's Balanced Salt Solution (HBSS) and suspended in 1x RPMI 1640 complete medium containing 10% Fetal Bovine Serum, Penicillin and Streptomycin until further use.

##### Serum

BD Vacutainer Serum tubes (Becton Dickinson) containing blood samples were allowed to clot and centrifuged to isolate serum. Serum samples were stored at −80°C until further use.

##### Total RNA isolation

About 5×10^6^ PBMCs freshly isolated at each time point (day 0, 2/4, 7 and 28) were suspended in Trizol (Life Technologies) and immediately stored at −80<sup>00BA</sup> C. Total RNA was isolated using the miRNeasy Mini Kit (Qiagen, CA) according to the manufacturer's instructions. Briefly, tubes containing PBMCs lysed with Trizol reagent was incubated at room temperature (15^0^C to 20^0^C) for 5 minutes. After chloroform extraction, the upper aqueous layer containing total RNA were collected to a clean sample tubes for processing using a QiaCube instrument (Qiagen, CA). The concentration of isolated total RNA was determined using the ND1000 Spectrophotometer, Nano Drop instrument. RNA samples were stored at −80°C until further use.

#### RNA Processing and Array Analysis

For gene expression analyses, samples were processed at the Yale Center for Genome Analysis (YCGA). Each RNA sample was quantified and integrity assessed by the Agilent 2100 BioAnalyser (Agilent, CA). Samples were processed for cRNA generation using the Illumina TotalPrep cRNA Amplification Kit and subsequently hybridized to the Human HT12-V4.0 BeadChip (Illumina, CA).

#### HAI Titer Measurement

Hemagglutination inhibition (HAI) assays were performed as described [32] on serum samples collected at days 0 and 28 after vaccination to determine antibody titers against each strain of influenza included in the 2010-2011 and 2011-2012 vaccines (A/California/7/09 (H1N1)-like virus; A/Perth /16/2009 (H3N2); and B/Brisbane/60/2008).

#### mtDNA content analysis

Total DNA was isolated from PBMCs using DNeasy kits (QIAGEN). A convenience sample of 7 older responders, 8 older non-responders, 6 young responders and 5 young non-responders was evaluated. Samples were adjusted to 1 ng/μl final concentration and subjected to SYBR Green-based real-time qPCR analysis of both nuclear and mitochondrial DNA content as described in Venegas et al. [33]. Biological samples were analyzed in triplicate: day 0 samples were centered at 1, and day 2, 7, and 28 samples were displayed as fold change relative to day 0. A t-test was used to evaluate statistical significance. The following primers were utilized for qPCR:

mtDNA tRNALeu Fwd: CACCCAAGAACAGGGTTTGT

mtDNA tRNALeu Rev: TGGCCATGGGTATGTTGTTA

nDNA β2-microglobulin Fwd: TGCTGTCTCCATGTTTGATGTATCT

nDNA β2-microglobulin Rev: TCTCTGCTCCCCACCTCTAAGT

#### Western Blot Analysis

Approximately 1× 10^6^ PBMCs were treated with cell lysis buffer (Cell Signaling Technology) from a subset of 5 young responders, 5 young non-responders, 5 older responders, and 5 older non-responders for which sufficient cell amounts were available. Lysed samples were briefly sonicated, and kept on ice for an additional 10 minutes. Lysates were mixed with 2x Laemmli Sample buffer, boiled and loaded onto 4-20% mini protean ready gels (BioRad). Following transfer, membranes were blocked with 5% milk powder in 1X TBS-tween 20 buffer at room temperature for 1 hour. Blocked membranes were incubated with primary antibodies against human HSP60 (Cell Signaling Technology), Succinate Dehydrogenase (SDHA) (Cell Signaling Technology) and HRP conjugated beta actin (anti-human beta-actin, Sigma), following by an HRP conjugated anti-Rabbit secondary antibody (Cell Signaling Technology) in 1x TBS-tween 20 containing 5% milk powder. Membranes were washed and developed and pixel intensities of the specific protein band and corresponding actin band on autoradiographs were estimated using ImageJ software. The relative pixel intensities (signal/actin) were plotted and statistical significance evaluated using a t-test.

#### Microarray analysis

RNA samples were processed and hybridized to HumanHT-12v4 Expression BeadChip (Illumina San Diego, CA), NCBI RefSeq Release 38 (November 7, 2009) and selected from GenBank, dbEST, and RefSeq. Arrays were processed at Yale's Keck Biotechnology Resource Laboratory and raw expression data were output by the Illumina GenomeStudio software. Microarray data are available through the Gene Expression Omnibus (GEO) Database, accession number GSE59635 and GSE59654. The data were Quantile normalized. Genes with multiple probes were collapsed to keep genes (official gene symbol) with highest average expression within differentially expressed genes. Pathway activity was calculated using QuSage [34] upon collapsing multiple probe ids mapping to a single official gene symbol. Differential expression was defined for each gene at each post-vaccination time-point using two criteria: (1) an absolute fold-change of at least 1.25 relative to pre-vaccination time point and (2) a significant change in expression by LIMMA (BioConductor (doi: 10.1186/gb-2004-5-10-r80.) implementation) after correction for multiple hypothesis testing (q <0.05). Differentially expressed genes were grouped into two clusters using hierarchical clustering by ward. All of this analysis was performed using BioConductor software packages (doi: 10.1186/gb-2004-5-10-r80) in R.

#### Transcription factor target identification

Using the UCSC Genome Bioinformatics site, we downloaded the transcription start site data (TSS) for all human RefSeq genes, defined by the January 2010 refGene table (doi: 10.1093/nar/gki476). The region +/−2Kb around each TSS was identified within a genome-wide multiple alignment of 45 vertebrate species to the human genome (doi: 10.1101/gr.1933104), also available through the UCSC Genome Bioinformatics site. In order to identify putative transcription factor binding sites, the human sequences, along with aligned regions from mouse, were masked for repetitive elements using RepeatMasker (http://www.repeatmasker.org) and then analyzed using the TRANSFAC MATCH (doi: 10.1093/nar/gkg585) algorithm with a cutoff, as defined within the database, chosen to minimize the sum of false positives and false negatives. The analysis was performed for all high quality vertebrate transcription factor matrices in the 2011.1 release of TRANSFAC (doi: 10.1093/nar/gkg108), and putative binding sites were considered to be evolutionarily conserved if matches were also found at the aligned positions in the mouse sequences and had no gaps present in the multiple alignment between the species being compared. Each TRANSFAC matrix was linked to a set of gene symbols describing potential binding factors using annotations present in the "Binding Factor" field of the database. Only vertebrate TRANSFAC matrices that could be linked to a HGNC gene symbol, either directly or through an alias listed in NCBI gene, were included.

## SUPPLEMENTAL DATA



## 



## 



## 



## 



## 



## 


