# Supplementary material for: Aging-dependent alterations in gene expression and a mitochondrial signature of responsiveness to human influenza vaccination
Source: Aging (Albany NY). 2015 Jan 14;7(1):38–51. doi: 10.18632/aging.100720 (PMC4356402; doi:10.18632/aging.100720)
Supplement: Supplementary file 2 [file aging-07-38-s002.doc]

Supplementary Table 2: Differential expression analysis of pre-vaccination transcriptional profiles in young vs. older adults revealing genes up-regulated (q<0.05 and |Fold-Change|≥1.25) in young and older adults.

| **Genes up-regulated in young adults** | **Genes up-regulated in older adults** |  |  |
| --- | --- | --- | --- |
| AAK1 | ARL6IP1 |  |  |
| AGAP8 | GBP5 |  |  |
| ALDOB | ITGB1 |  |  |
| ARRDC5 | P2RY5 |  |  |
| CD248 | REEP5 |  |  |
| CSAD |  |  |  |
| EP300 |  |  |  |
| FCGBP |  |  |  |
| GTPBP3 |  |  |  |
| IQSEC1 |  |  |  |
| LOC100132247 |  |  |  |
| LOC100190986 |  |  |  |
| LOC220686 |  |  |  |
| LOC285359 |  |  |  |
| LOC338799 |  |  |  |
| LOC440341 |  |  |  |
| LOC440348 |  |  |  |
| LOC613037 |  |  |  |
| LOC644931 |  |  |  |
| LOC653316 |  |  |  |
| LOC728734 |  |  |  |
| LOC728888 |  |  |  |
| LRRN3 |  |  |  |
| NARG1L |  |  |  |
| NPIP |  |  |  |
| NSUN5B |  |  |  |
| PDE7A |  |  |  |
| PI4KAP1 |  |  |  |
| PI4KAP2 |  |  |  |
| PPTC7 |  |  |  |
| RAI1 |  |  |  |
| RNU4-1 |  |  |  |
| SNORD4A |  |  |  |
| SREBF1 |  |  |  |
| ZCCHC11 |  |  |  |
|  |  |  |  |
|  |  |  |  |
|  |  |  |  |
|  |  |  |  |
|  |  |  |  |
