# Supplementary material for: Aging-dependent alterations in gene expression and a mitochondrial signature of responsiveness to human influenza vaccination
Source: Aging (Albany NY). 2015 Jan 14;7(1):38–51. doi: 10.18632/aging.100720 (PMC4356402; doi:10.18632/aging.100720)
Supplement: Supplementary file 4 [file aging-07-38-s004.doc]

Supplementary table 3B: False Discovery Rates (FDRs) associated with vaccination-induced activities (FDR < 0.05 for at least one of the condition) for the Blood Transcriptional Modules (BTMs) defined in . QuSAGE was used to obtain P values for detecting the activity of each module by comparing pre- and post-vaccination transcriptional profiles. FDRs were obtained using the method of Benjamini and Hochberg .

|  | Young | | | | | | Older | | | | | | Frail | | |
| --- | --- | --- | --- | --- | --- | --- | --- | --- | --- | --- | --- | --- | --- | --- | --- |
|  | R+ | R+ | R+ | R- | R- | R- | R+ | R+ | R+ | R- | R- | R- | R- | R- | R- |
| *Days post-vaccination* | 2 | 7 | 28 | 2 | 7 | 28 | 2 | 7 | 28 | 2 | 7 | 28 | 2 | 7 | 28 |
| Activated (LPS) dendritic cell surface signature (S11) | 9.4E-01 | 8.2E-01 | 8.1E-01 | 5.9E-01 | 9.7E-01 | 6.1E-01 | 8.6E-01 | 8.9E-01 | 3.0E-01 | 1.5E-03 | 5.6E-05 | 1.5E-02 | 1.0E+00 | 9.9E-01 | 1.0E+00 |
| activated dendritic cells (M67) | 7.0E-01 | 6.0E-02 | 9.6E-01 | 4.6E-01 | 6.7E-01 | 2.5E-01 | 4.7E-01 | 8.0E-01 | 7.2E-01 | 8.9E-02 | 3.0E-01 | 1.2E-02 | 1.0E+00 | 9.9E-01 | 1.0E+00 |
| antigen presentation (lipids and proteins) (M28) | 9.4E-01 | 3.0E-02 | 2.5E-02 | 9.6E-01 | 5.2E-01 | 1.2E-01 | 3.1E-01 | 3.7E-01 | 9.8E-01 | 1.0E-04 | 1.2E-02 | 1.2E-02 | 1.0E+00 | 9.9E-01 | 1.0E+00 |
| antigen processing and presentation (M200) | 7.3E-01 | 9.0E-02 | 4.5E-03 | 8.6E-01 | 4.6E-01 | 1.0E+00 | 2.6E-01 | 1.9E-01 | 9.4E-01 | 2.0E-04 | 2.2E-04 | 6.6E-04 | 1.0E+00 | 9.9E-01 | 1.0E+00 |
| antiviral IFN signature (M75) | 9.7E-01 | 1.2E-03 | 1.8E-03 | 6.1E-01 | 5.7E-02 | 4.4E-03 | 2.7E-01 | 9.4E-01 | 7.7E-01 | 1.1E-03 | 2.7E-02 | 7.7E-01 | 1.0E+00 | 9.9E-01 | 1.0E+00 |
| AP-1 transcription factor network (M20) | 7.0E-01 | 9.6E-04 | 1.6E-01 | 8.7E-01 | 7.9E-01 | 1.4E-01 | 1.5E-01 | 3.4E-01 | 8.1E-01 | 4.3E-02 | 3.8E-03 | 4.1E-01 | 1.0E+00 | 9.9E-01 | 1.0E+00 |
| axon guidance (M110) | 7.1E-01 | 9.4E-01 | 6.7E-01 | 1.2E-01 | 9.6E-01 | 9.8E-01 | 3.7E-01 | 4.9E-01 | 7.9E-01 | 3.8E-01 | 4.8E-02 | 6.3E-02 | 1.0E+00 | 1.0E+00 | 1.0E+00 |
| B cell development (M9) | 1.0E+00 | 1.6E-02 | 5.9E-02 | 9.2E-01 | 2.6E-01 | 3.8E-01 | 5.0E-01 | 7.9E-01 | 7.0E-01 | 2.3E-03 | 2.4E-01 | 1.4E-01 | 1.0E+00 | 9.9E-01 | 1.0E+00 |
| B cell development/activation (M58) | 9.4E-01 | 6.6E-01 | 2.9E-01 | 7.6E-01 | 5.8E-02 | 2.2E-02 | 7.4E-01 | 7.9E-01 | 5.2E-01 | 7.0E-01 | 1.4E-02 | 4.6E-02 | 1.0E+00 | 9.9E-01 | 1.0E+00 |
| B cell surface signature (S2) | 9.9E-01 | 3.1E-01 | 2.2E-01 | 2.0E-02 | 5.1E-05 | 6.6E-06 | 2.0E-01 | 3.7E-01 | 1.0E+00 | 1.3E-02 | 5.2E-04 | 2.0E-01 | 1.0E+00 | 9.9E-01 | 1.0E+00 |
| BCR signaling (M54) | 9.9E-01 | 1.6E-02 | 7.7E-03 | 7.6E-01 | 9.7E-01 | 9.8E-01 | 2.6E-01 | 6.2E-01 | 8.1E-01 | 5.9E-04 | 6.0E-01 | 2.4E-02 | 1.0E+00 | 9.9E-01 | 1.0E+00 |
| blood coagulation (M11.1) | 7.0E-01 | 9.6E-04 | 1.1E-03 | 6.9E-02 | 2.2E-01 | 2.3E-03 | 1.1E-01 | 8.9E-01 | 5.1E-01 | 1.5E-07 | 1.9E-07 | 1.5E-07 | 1.0E+00 | 9.9E-01 | 1.0E+00 |
| CD1 and other DC receptors (M50) | 7.0E-01 | 1.5E-02 | 7.3E-03 | 1.1E-01 | 8.7E-01 | 3.5E-01 | 4.5E-01 | 9.4E-01 | 8.1E-01 | 1.1E-03 | 3.6E-02 | 7.8E-03 | 1.0E+00 | 9.9E-01 | 1.0E+00 |
| CD28 costimulation (M12) | 7.0E-01 | 3.8E-03 | 1.7E-03 | 9.1E-01 | 1.5E-01 | 8.9E-03 | 4.1E-01 | 9.4E-01 | 9.7E-01 | 6.4E-04 | 2.3E-02 | 3.7E-04 | 1.0E+00 | 9.9E-01 | 1.0E+00 |
| CD4 T cell surface signature Th1-stimulated (S6) | 8.5E-01 | 3.0E-01 | 6.9E-01 | 1.1E-01 | 1.6E-02 | 7.8E-04 | 7.4E-01 | 8.6E-01 | 7.0E-01 | 2.8E-02 | 2.4E-02 | 5.0E-04 | 1.0E+00 | 9.9E-01 | 1.0E+00 |
| CD4 T cell surface signature Th2-stimulated (S7) | 8.6E-01 | 2.2E-02 | 6.5E-03 | 9.3E-01 | 9.7E-01 | 6.7E-01 | 7.3E-01 | 3.4E-01 | 3.7E-01 | 7.4E-04 | 1.2E-01 | 5.8E-03 | 1.0E+00 | 9.9E-01 | 1.0E+00 |
| cell activation (IL15, IL23, TNF) (M24) | 7.1E-01 | 1.9E-03 | 1.3E-02 | 3.9E-01 | 1.3E-03 | 7.3E-04 | 1.2E-01 | 8.9E-01 | 8.1E-01 | 1.8E-04 | 7.7E-01 | 1.3E-02 | 1.0E+00 | 9.9E-01 | 1.0E+00 |
| cell adhesion (GO) (M117) | 9.6E-01 | 1.4E-03 | 1.2E-02 | 6.2E-01 | 7.5E-05 | 1.6E-04 | 1.7E-02 | 6.5E-01 | 5.1E-01 | 1.1E-03 | 1.4E-02 | 3.4E-01 | 1.0E+00 | 9.9E-01 | 1.0E+00 |
| cell adhesion (lymphocyte homing) (M21) | 9.4E-01 | 4.7E-01 | 8.3E-01 | 4.8E-01 | 2.8E-01 | 3.0E-02 | 9.1E-01 | 6.5E-01 | 9.4E-01 | 7.6E-01 | 2.7E-02 | 3.1E-02 | 1.0E+00 | 9.9E-01 | 1.0E+00 |
| cell adhesion (M51) | 6.4E-01 | 3.3E-01 | 5.1E-03 | 4.5E-02 | 9.7E-01 | 3.0E-02 | 7.3E-01 | 9.4E-01 | 9.4E-01 | 2.5E-02 | 5.4E-06 | 1.9E-05 | 1.0E+00 | 9.9E-01 | 1.0E+00 |
| cell cell adhesion (M133.1) | 7.1E-01 | 6.7E-01 | 2.3E-01 | 6.1E-01 | 6.7E-01 | 6.9E-01 | 7.0E-01 | 9.3E-01 | 7.2E-01 | 2.7E-02 | 5.2E-01 | 7.6E-02 | 1.0E+00 | 9.9E-01 | 1.0E+00 |
| cell cycle (I) (M4.1) | 6.4E-01 | 5.5E-05 | 4.5E-04 | 9.0E-01 | 3.4E-02 | 1.6E-02 | 1.2E-01 | 1.4E-01 | 9.1E-02 | 7.0E-11 | 5.2E-03 | 9.9E-07 | 1.0E+00 | 9.9E-01 | 1.0E+00 |
| cell cycle (II) (M4.10) | 8.0E-01 | 2.0E-02 | 3.3E-01 | 8.7E-01 | 5.9E-01 | 8.8E-01 | 7.4E-01 | 1.8E-01 | 2.1E-01 | 1.1E-01 | 9.9E-01 | 7.7E-01 | 1.0E+00 | 9.9E-01 | 1.0E+00 |
| cell cycle (III) (M103) | 9.7E-01 | 1.9E-03 | 1.2E-02 | 6.9E-01 | 4.9E-01 | 2.6E-02 | 8.7E-01 | 4.9E-01 | 4.4E-01 | 3.0E-03 | 1.8E-01 | 7.8E-03 | 1.0E+00 | 9.9E-01 | 1.0E+00 |
| cell cycle and growth arrest (M31) | 6.4E-01 | 2.6E-04 | 3.0E-02 | 4.4E-01 | 1.2E-01 | 1.6E-03 | 3.3E-02 | 7.9E-01 | 9.5E-01 | 4.0E-05 | 8.8E-01 | 2.3E-03 | 1.0E+00 | 9.9E-01 | 1.0E+00 |
| cell cycle and transcription (M4.0) | 7.1E-01 | 3.0E-03 | 3.7E-05 | 1.1E-04 | 4.3E-04 | 1.6E-08 | 1.7E-02 | 4.6E-01 | 3.3E-01 | 3.4E-12 | 4.5E-12 | 5.6E-12 | 1.0E+00 | 9.9E-01 | 1.0E+00 |
| cell cycle, ATP binding (M144) | 8.8E-01 | 4.1E-03 | 7.4E-04 | 1.1E-01 | 1.6E-02 | 5.4E-05 | 3.7E-01 | 2.6E-01 | 8.1E-01 | 3.5E-06 | 1.8E-01 | 3.0E-03 | 1.0E+00 | 6.3E-01 | 1.0E+00 |
| cell cycle, mitotic phase (M230) | 9.7E-01 | 2.4E-03 | 1.6E-03 | 3.7E-01 | 2.9E-04 | 1.3E-04 | 6.2E-01 | 3.7E-01 | 8.1E-01 | 5.9E-04 | 8.1E-01 | 4.4E-03 | 1.0E+00 | 7.4E-01 | 1.0E+00 |
| cell division (stimulated CD4+ T cells) (M46) | 9.7E-01 | 1.9E-01 | 9.8E-01 | 2.1E-01 | 1.0E+00 | 8.9E-01 | 2.7E-01 | 5.6E-01 | 3.7E-01 | 4.4E-02 | 9.9E-02 | 6.0E-02 | 1.0E+00 | 9.9E-01 | 1.0E+00 |
| cell division in stimulated CD4 T cells (M4.6) | 6.4E-01 | 3.0E-03 | 8.0E-02 | 5.6E-01 | 6.2E-01 | 7.3E-01 | 6.7E-01 | 4.0E-01 | 2.2E-01 | 1.4E-04 | 1.0E-03 | 3.0E-03 | 1.0E+00 | 9.9E-01 | 1.0E+00 |
| cell junction (GO) (M4.13) | 8.7E-01 | 2.7E-03 | 1.7E-03 | 3.3E-01 | 4.3E-01 | 1.5E-02 | 8.6E-02 | 7.8E-01 | 4.1E-01 | 1.4E-07 | 2.6E-05 | 4.7E-06 | 1.0E+00 | 9.9E-01 | 1.0E+00 |
| cell junction (M162.1) | 7.1E-01 | 2.6E-02 | 1.2E-01 | 8.7E-01 | 9.7E-01 | 9.2E-01 | 7.6E-01 | 8.9E-01 | 9.0E-01 | 1.2E-01 | 3.3E-01 | 3.7E-01 | 1.0E+00 | 9.9E-01 | 1.0E+00 |
| cell movement, Adhesion & Platelet activation (M30) | 6.4E-01 | 4.8E-02 | 5.4E-03 | 6.9E-02 | 8.7E-01 | 8.6E-04 | 9.9E-01 | 9.6E-01 | 7.7E-01 | 2.6E-01 | 1.6E-04 | 3.6E-05 | 1.0E+00 | 9.9E-01 | 1.0E+00 |
| chaperonin mediated protein folding (I) (M204.0) | 6.4E-01 | 6.8E-04 | 5.6E-04 | 9.2E-02 | 8.6E-04 | 1.9E-03 | 7.8E-02 | 1.4E-01 | 9.2E-01 | 3.1E-07 | 1.1E-01 | 1.1E-04 | 1.0E+00 | 7.4E-01 | 1.0E+00 |
| chaperonin mediated protein folding (II) (M204.1) | 7.0E-01 | 6.8E-04 | 6.2E-04 | 5.2E-02 | 5.2E-05 | 1.6E-05 | 4.6E-02 | 3.4E-01 | 9.4E-01 | 5.8E-07 | 7.9E-01 | 7.6E-04 | 1.0E+00 | 4.2E-01 | 1.0E+00 |
| chemokine cluster (II) (M27.1) | 9.9E-01 | 9.7E-01 | 4.6E-01 | 2.8E-01 | 5.9E-02 | 9.8E-01 | 5.3E-01 | 6.5E-01 | 9.5E-01 | 6.7E-01 | 2.2E-04 | 6.0E-01 | 1.0E+00 | 9.9E-01 | 1.0E+00 |
| chemokines and inflammatory molecules in myeloid cells (M86.0) | 7.1E-01 | 7.0E-04 | 3.2E-02 | 5.9E-01 | 1.1E-01 | 5.5E-03 | 3.4E-02 | 6.5E-01 | 9.4E-01 | 2.2E-03 | 4.7E-02 | 3.7E-01 | 1.0E+00 | 9.9E-01 | 1.0E+00 |
| chemokines and receptors (M38) | 7.2E-01 | 1.0E-03 | 1.7E-01 | 6.0E-01 | 4.3E-01 | 1.9E-01 | 1.9E-01 | 9.3E-01 | 9.4E-01 | 7.7E-03 | 7.7E-01 | 2.0E-01 | 1.0E+00 | 9.9E-01 | 1.0E+00 |
| C-MYC transcriptional network (M4.12) | 9.3E-01 | 2.1E-03 | 1.9E-02 | 8.7E-01 | 1.8E-01 | 2.3E-01 | 3.9E-01 | 3.4E-01 | 7.0E-01 | 1.4E-03 | 4.3E-01 | 1.6E-01 | 1.0E+00 | 9.9E-01 | 1.0E+00 |
| collagen, TGFB family et al (M77) | 7.3E-01 | 7.7E-01 | 8.3E-01 | 3.9E-01 | 8.8E-01 | 2.9E-01 | 4.7E-01 | 7.8E-01 | 9.2E-01 | 2.9E-01 | 2.4E-02 | 2.4E-02 | 1.0E+00 | 9.9E-01 | 1.0E+00 |
| complement activation (I) (M112.0) | 9.9E-01 | 3.0E-03 | 5.8E-02 | 8.7E-01 | 6.5E-02 | 5.1E-02 | 8.5E-01 | 7.9E-01 | 7.0E-01 | 9.1E-03 | 3.3E-02 | 5.8E-02 | 1.0E+00 | 9.9E-01 | 1.0E+00 |
| complement and other receptors in DCs (M40) | 9.4E-01 | 9.8E-02 | 2.8E-02 | 8.4E-01 | 3.3E-01 | 5.3E-01 | 8.7E-01 | 7.8E-01 | 3.9E-01 | 8.4E-05 | 5.4E-05 | 1.7E-02 | 1.0E+00 | 9.9E-01 | 1.0E+00 |
| CORO1A-DEF6 network (I) (M32.2) | 9.9E-01 | 9.6E-04 | 1.3E-03 | 4.0E-01 | 5.2E-05 | 2.8E-05 | 1.7E-02 | 6.5E-01 | 4.3E-01 | 3.2E-07 | 1.4E-03 | 4.2E-02 | 1.0E+00 | 9.9E-01 | 1.0E+00 |
| CORO1A-DEF6 network (II) (M32.4) | 9.7E-01 | 1.7E-01 | 1.4E-02 | 6.7E-01 | 3.9E-01 | 5.4E-02 | 4.1E-01 | 4.0E-01 | 9.8E-01 | 2.8E-04 | 1.7E-02 | 3.4E-01 | 1.0E+00 | 9.9E-01 | 1.0E+00 |
| cytokines - recepters cluster (M115) | 9.4E-01 | 9.4E-01 | 4.4E-01 | 8.7E-01 | 3.5E-01 | 7.4E-01 | 5.6E-01 | 8.9E-01 | 9.5E-01 | 1.2E-02 | 9.8E-02 | 1.3E-01 | 1.0E+00 | 9.9E-01 | 1.0E+00 |
| cytoskeletal remodeling (enriched for SRF targets) (M34) | 6.4E-01 | 9.2E-01 | 1.7E-02 | 3.2E-01 | 9.7E-01 | 2.1E-02 | 7.9E-01 | 8.9E-01 | 9.0E-01 | 2.3E-02 | 5.2E-04 | 4.4E-04 | 1.0E+00 | 9.9E-01 | 1.0E+00 |
| cytoskeletal remodeling (M32.8) | 7.2E-01 | 9.6E-04 | 1.4E-03 | 1.3E-01 | 3.9E-04 | 1.4E-03 | 3.4E-02 | 4.9E-01 | 7.2E-01 | 6.6E-05 | 4.7E-01 | 2.3E-02 | 1.0E+00 | 1.0E+00 | 1.0E+00 |
| cytoskeleton/actin (SRF transcription targets) (M145.0) | 6.4E-01 | 6.1E-01 | 8.2E-02 | 1.9E-01 | 6.2E-01 | 3.8E-01 | 7.0E-01 | 1.0E+00 | 9.0E-01 | 6.9E-02 | 1.1E-02 | 4.9E-03 | 1.0E+00 | 9.9E-01 | 1.0E+00 |
| cytoskeleton/actin (SRF transcription targets) (M145.1) | 7.0E-01 | 5.0E-01 | 3.9E-03 | 8.1E-02 | 4.0E-01 | 4.8E-02 | 9.4E-01 | 7.8E-01 | 9.4E-01 | 1.4E-01 | 9.1E-03 | 1.4E-03 | 1.0E+00 | 9.9E-01 | 1.0E+00 |
| DC surface signature (S5) | 7.1E-01 | 8.3E-01 | 6.6E-01 | 5.4E-01 | 4.2E-02 | 2.8E-02 | 8.5E-01 | 7.8E-01 | 6.9E-01 | 3.3E-04 | 7.1E-07 | 2.8E-06 | 1.0E+00 | 9.9E-01 | 1.0E+00 |
| DNA repair (M76) | 9.0E-01 | 1.0E-02 | 7.1E-03 | 4.8E-01 | 2.3E-01 | 4.4E-02 | 2.9E-01 | 4.9E-01 | 7.0E-01 | 1.4E-05 | 2.2E-01 | 3.1E-02 | 1.0E+00 | 9.9E-01 | 1.0E+00 |
| double positive thymocytes (M126) | 8.0E-01 | 1.3E-01 | 1.3E-02 | 9.2E-01 | 5.5E-01 | 9.2E-01 | 7.4E-01 | 8.3E-01 | 7.2E-01 | 7.9E-01 | 5.8E-01 | 2.4E-01 | 1.0E+00 | 1.0E+00 | 1.0E+00 |
| E2F1 targets (Q3) (M10.0) | 9.7E-01 | 2.3E-02 | 4.9E-02 | 7.3E-01 | 9.7E-01 | 5.8E-02 | 7.3E-01 | 6.8E-01 | 3.7E-01 | 2.5E-01 | 9.3E-01 | 4.4E-01 | 1.0E+00 | 9.9E-01 | 1.0E+00 |
| E2F1 targets (Q4) (M10.1) | 7.0E-01 | 9.6E-04 | 9.3E-03 | 3.9E-01 | 1.2E-01 | 4.5E-02 | 9.1E-01 | 3.0E-01 | 3.1E-01 | 9.5E-05 | 6.1E-01 | 5.6E-03 | 1.0E+00 | 9.9E-01 | 1.0E+00 |
| endoplasmic reticulum (M37.2) | 7.2E-01 | 3.0E-03 | 2.5E-02 | 7.8E-01 | 9.7E-01 | 4.4E-01 | 3.8E-01 | 9.5E-01 | 5.1E-01 | 2.7E-04 | 4.3E-03 | 7.6E-03 | 1.0E+00 | 9.9E-01 | 1.0E+00 |
| enriched for cell migration (M122) | 9.7E-01 | 7.2E-01 | 4.5E-01 | 6.9E-01 | 3.9E-01 | 9.3E-01 | 8.6E-01 | 8.9E-01 | 9.4E-01 | 5.2E-01 | 2.4E-02 | 2.4E-01 | 1.0E+00 | 9.9E-01 | 1.0E+00 |
| enriched for promoter motif NATCACGTGAY (putative SREBF1 targets) (M178) | 8.0E-01 | 2.3E-02 | 1.4E-02 | 6.9E-01 | 4.3E-01 | 1.3E-02 | 4.7E-01 | 8.4E-01 | 9.4E-01 | 8.4E-02 | 8.5E-01 | 7.7E-01 | 1.0E+00 | 9.9E-01 | 1.0E+00 |
| enriched for SMAD2/3 signaling (M97) | 7.3E-01 | 2.2E-01 | 1.2E-01 | 4.1E-01 | 4.5E-02 | 1.4E-02 | 1.2E-01 | 8.9E-01 | 3.9E-01 | 3.0E-01 | 6.9E-01 | 9.6E-01 | 1.0E+00 | 9.9E-01 | 1.0E+00 |
| enriched for TF motif PAX3 (M179) | 9.7E-01 | 4.7E-03 | 1.4E-03 | 2.1E-01 | 7.1E-03 | 4.8E-04 | 1.9E-01 | 4.0E-01 | 7.2E-01 | 1.2E-05 | 2.6E-01 | 1.6E-03 | 1.0E+00 | 7.6E-01 | 1.0E+00 |
| enriched for TF motif TNCATNTCCYR (M232) | 9.7E-01 | 1.9E-03 | 6.6E-03 | 9.1E-01 | 7.3E-03 | 2.1E-02 | 3.0E-01 | 1.4E-01 | 9.9E-01 | 7.7E-03 | 1.0E+00 | 1.2E-02 | 1.0E+00 | 7.4E-01 | 1.0E+00 |
| enriched for TF motif TTCNRGNNNNTTC (M172) | 7.5E-01 | 1.4E-02 | 9.2E-02 | 5.8E-01 | 7.8E-01 | 1.9E-01 | 3.7E-01 | 9.8E-01 | 9.2E-01 | 2.9E-01 | 4.2E-01 | 2.7E-01 | 1.0E+00 | 9.9E-01 | 1.0E+00 |
| enriched for ubiquitination (M138) | 6.4E-01 | 2.6E-02 | 1.7E-03 | 8.8E-02 | 1.3E-03 | 2.2E-04 | 4.3E-02 | 5.6E-01 | 9.4E-01 | 2.9E-05 | 2.0E-01 | 2.8E-02 | 1.0E+00 | 9.9E-01 | 1.0E+00 |
| enriched in activated dendritic cells (I) (M119) | 8.0E-01 | 2.4E-02 | 8.9E-02 | 5.8E-01 | 5.7E-01 | 7.3E-02 | 6.3E-01 | 8.7E-01 | 7.3E-01 | 1.3E-04 | 5.9E-03 | 1.4E-03 | 1.0E+00 | 9.9E-01 | 1.0E+00 |
| enriched in activated dendritic cells (II) (M165) | 9.8E-01 | 4.5E-01 | 4.2E-01 | 7.7E-01 | 3.0E-02 | 6.0E-01 | 4.6E-01 | 5.6E-01 | 3.0E-01 | 6.2E-01 | 7.1E-03 | 1.0E-01 | 1.0E+00 | 1.0E+00 | 1.0E+00 |
| enriched in activated dendritic cells/monocytes (M64) | 7.0E-01 | 6.8E-04 | 2.7E-02 | 4.6E-01 | 9.1E-01 | 1.9E-02 | 1.2E-01 | 5.7E-01 | 2.2E-01 | 1.3E-05 | 9.0E-03 | 4.0E-04 | 1.0E+00 | 9.9E-01 | 1.0E+00 |
| enriched in antigen presentation (I) (M71) | 7.2E-01 | 9.6E-04 | 3.6E-03 | 9.6E-01 | 3.9E-01 | 6.6E-02 | 4.7E-02 | 3.7E-01 | 9.4E-01 | 2.5E-06 | 1.4E-04 | 7.6E-04 | 1.0E+00 | 9.9E-01 | 1.0E+00 |
| enriched in antigen presentation (II) (M95.0) | 8.0E-01 | 1.2E-03 | 1.6E-03 | 7.6E-01 | 1.6E-02 | 1.1E-02 | 1.7E-02 | 8.5E-01 | 9.5E-01 | 3.2E-07 | 1.7E-02 | 4.6E-02 | 1.0E+00 | 9.9E-01 | 1.0E+00 |
| enriched in antigen presentation (III) (M95.1) | 8.0E-01 | 4.8E-02 | 2.0E-02 | 8.5E-01 | 7.1E-01 | 6.0E-01 | 4.7E-02 | 4.9E-01 | 9.7E-01 | 2.7E-05 | 6.1E-03 | 1.3E-02 | 1.0E+00 | 9.9E-01 | 1.0E+00 |
| enriched in B cell differentiation (M123) | 7.0E-01 | 1.3E-02 | 3.9E-01 | 8.4E-01 | 4.2E-01 | 9.2E-01 | 4.9E-01 | 8.9E-01 | 8.8E-01 | 4.8E-01 | 1.8E-01 | 9.3E-01 | 1.0E+00 | 9.9E-01 | 1.0E+00 |
| enriched in B cells (I) (M47.0) | 8.0E-01 | 4.9E-02 | 3.3E-02 | 4.3E-05 | 2.5E-06 | 1.2E-06 | 1.2E-02 | 2.1E-02 | 9.5E-01 | 7.5E-07 | 4.2E-08 | 3.5E-06 | 1.0E+00 | 7.6E-01 | 1.0E+00 |
| enriched in B cells (II) (M47.1) | 8.4E-01 | 4.5E-03 | 8.2E-02 | 2.7E-03 | 8.4E-06 | 4.3E-06 | 1.7E-02 | 1.4E-01 | 9.6E-01 | 4.6E-05 | 1.6E-06 | 3.0E-04 | 1.0E+00 | 7.6E-01 | 1.0E+00 |
| enriched in B cells (III) (M47.2) | 9.7E-01 | 2.2E-02 | 8.1E-02 | 4.4E-03 | 5.2E-05 | 1.6E-05 | 2.5E-01 | 8.9E-01 | 9.4E-01 | 1.0E-01 | 6.5E-03 | 4.8E-02 | 1.0E+00 | 9.9E-01 | 1.0E+00 |
| enriched in B cells (IV) (M47.3) | 7.2E-01 | 7.5E-03 | 9.4E-03 | 9.2E-02 | 8.2E-04 | 1.5E-04 | 7.1E-02 | 9.0E-01 | 8.1E-01 | 2.7E-02 | 9.6E-01 | 7.4E-01 | 1.0E+00 | 9.9E-01 | 1.0E+00 |
| enriched in B cells (V) (M47.4) | 9.6E-01 | 7.0E-01 | 2.7E-02 | 1.7E-01 | 4.0E-01 | 4.8E-02 | 7.7E-01 | 8.9E-01 | 7.3E-01 | 2.9E-01 | 6.1E-03 | 5.9E-02 | 1.0E+00 | 9.9E-01 | 1.0E+00 |
| enriched in B cells (VI) (M69) | 9.7E-01 | 9.7E-01 | 3.4E-01 | 1.7E-02 | 1.3E-03 | 4.5E-04 | 6.1E-02 | 3.6E-02 | 8.1E-01 | 6.6E-03 | 3.2E-05 | 7.8E-03 | 1.0E+00 | 7.9E-01 | 1.0E+00 |
| enriched in cell cycle (M167) | 7.0E-01 | 3.3E-01 | 1.4E-01 | 4.0E-01 | 2.6E-01 | 2.2E-01 | 6.1E-01 | 8.0E-02 | 5.1E-01 | 7.9E-01 | 1.6E-03 | 1.0E+00 | 1.0E+00 | 7.4E-01 | 1.0E+00 |
| enriched in dendritic cells (M168) | 7.0E-01 | 4.5E-01 | 7.1E-01 | 1.0E-01 | 7.9E-01 | 5.8E-01 | 6.6E-01 | 7.7E-01 | 6.8E-01 | 1.8E-04 | 3.6E-05 | 4.7E-05 | 1.0E+00 | 9.9E-01 | 1.0E+00 |
| enriched in DNA interacting proteins (M182) | 9.4E-01 | 1.0E-03 | 2.0E-03 | 4.8E-01 | 1.2E-03 | 1.7E-04 | 9.4E-01 | 4.0E-01 | 7.2E-01 | 9.7E-03 | 5.2E-01 | 1.1E-01 | 1.0E+00 | 9.9E-01 | 1.0E+00 |
| enriched in extracellular matrix & associated proteins (M202) | 8.4E-01 | 5.5E-01 | 3.4E-02 | 4.6E-01 | 6.8E-01 | 2.0E-01 | 9.6E-01 | 9.3E-01 | 9.7E-01 | 5.6E-01 | 2.5E-02 | 4.9E-02 | 1.0E+00 | 9.9E-01 | 1.0E+00 |
| enriched in G-protein coupled receptors (M130) | 9.7E-01 | 9.0E-01 | 6.1E-01 | 1.8E-01 | 2.5E-02 | 7.0E-01 | 8.1E-01 | 8.3E-01 | 9.4E-01 | 7.6E-02 | 1.0E-02 | 2.6E-03 | 1.0E+00 | 9.9E-01 | 1.0E+00 |
| enriched in monocytes (I) (M4.15) | 9.4E-01 | 3.1E-02 | 6.1E-03 | 3.0E-01 | 2.9E-01 | 2.1E-03 | 2.4E-01 | 4.6E-01 | 5.1E-01 | 1.5E-06 | 3.6E-05 | 2.6E-05 | 1.0E+00 | 9.9E-01 | 1.0E+00 |
| enriched in monocytes (II) (M11.0) | 6.4E-01 | 3.1E-06 | 3.3E-06 | 7.8E-06 | 2.5E-06 | 1.6E-08 | 3.9E-04 | 5.6E-01 | 3.9E-03 | 1.6E-12 | 2.2E-12 | 1.5E-12 | 1.0E+00 | 9.9E-01 | 1.0E+00 |
| enriched in monocytes (III) (M73) | 7.0E-01 | 1.3E-02 | 3.3E-01 | 6.2E-01 | 6.8E-01 | 4.1E-01 | 2.6E-01 | 9.0E-01 | 2.6E-01 | 2.2E-03 | 7.0E-03 | 2.6E-03 | 1.0E+00 | 9.9E-01 | 1.0E+00 |
| enriched in monocytes (IV) (M118.0) | 6.4E-01 | 2.1E-03 | 1.3E-02 | 1.6E-01 | 4.7E-02 | 1.0E-01 | 4.7E-02 | 6.0E-01 | 1.1E-01 | 2.1E-09 | 2.2E-10 | 2.8E-09 | 1.0E+00 | 9.9E-01 | 1.0E+00 |
| enriched in monocytes (surface) (M118.1) | 9.4E-01 | 2.2E-01 | 9.2E-03 | 4.1E-01 | 5.5E-01 | 1.5E-01 | 2.5E-01 | 8.9E-01 | 2.2E-01 | 1.9E-06 | 2.4E-05 | 3.8E-06 | 1.0E+00 | 9.9E-01 | 1.0E+00 |
| enriched in myeloid cells and monocytes (M81) | 6.4E-01 | 7.5E-01 | 7.4E-03 | 4.4E-02 | 9.7E-01 | 1.8E-01 | 4.7E-01 | 9.8E-01 | 4.1E-01 | 3.5E-06 | 1.0E-08 | 4.3E-08 | 1.0E+00 | 1.0E+00 | 1.0E+00 |
| enriched in naive and memory B cells (M83) | 7.2E-01 | 8.5E-01 | 6.5E-01 | 4.6E-01 | 1.1E-01 | 1.6E-01 | 7.0E-01 | 7.2E-01 | 9.4E-01 | 5.0E-01 | 6.7E-01 | 3.4E-02 | 1.0E+00 | 9.9E-01 | 1.0E+00 |
| enriched in neutrophils (I) (M37.1) | 7.1E-01 | 6.8E-04 | 4.7E-03 | 6.0E-01 | 4.5E-03 | 3.9E-01 | 4.2E-02 | 1.4E-01 | 1.2E-02 | 2.4E-05 | 1.5E-01 | 1.7E-03 | 1.0E+00 | 9.9E-01 | 1.0E+00 |
| enriched in neutrophils (II) (M163) | 7.1E-01 | 1.2E-03 | 5.5E-03 | 2.8E-01 | 7.0E-01 | 1.1E-01 | 4.7E-01 | 3.7E-01 | 3.0E-01 | 6.5E-05 | 2.2E-02 | 6.3E-03 | 1.0E+00 | 9.9E-01 | 1.0E+00 |
| enriched in NK cells (I) (M7.2) | 7.0E-01 | 6.8E-04 | 5.4E-01 | 2.4E-03 | 1.3E-03 | 3.9E-01 | 7.1E-02 | 8.4E-01 | 4.4E-01 | 8.3E-09 | 2.4E-07 | 5.1E-09 | 1.0E+00 | 1.0E+00 | 1.0E+00 |
| enriched in NK cells (II) (M61.0) | 9.7E-01 | 7.5E-01 | 1.2E-01 | 2.3E-02 | 5.1E-05 | 6.4E-03 | 8.6E-01 | 6.1E-01 | 5.6E-01 | 4.3E-01 | 3.9E-02 | 1.3E-06 | 1.0E+00 | 9.9E-01 | 1.0E+00 |
| enriched in NK cells (III) (M157) | 9.4E-01 | 6.0E-01 | 9.4E-01 | 4.6E-01 | 2.7E-02 | 9.2E-01 | 5.5E-01 | 7.8E-01 | 8.3E-01 | 6.7E-03 | 4.8E-02 | 1.6E-05 | 1.0E+00 | 9.9E-01 | 1.0E+00 |
| enriched in NK cells (KIR cluster) (M61.1) | 9.4E-01 | 5.4E-02 | 3.6E-02 | 3.1E-01 | 5.4E-04 | 3.2E-02 | 4.9E-01 | 5.1E-01 | 9.7E-01 | 7.4E-01 | 3.0E-01 | 2.1E-02 | 1.0E+00 | 9.9E-01 | 1.0E+00 |
| enriched in NK cells (receptor activation) (M61.2) | 9.7E-01 | 1.5E-01 | 3.6E-02 | 2.3E-01 | 8.2E-03 | 5.1E-02 | 6.6E-01 | 6.8E-01 | 5.1E-01 | 7.3E-01 | 2.2E-02 | 1.4E-03 | 1.0E+00 | 9.9E-01 | 1.0E+00 |
| enriched in nuclear pore complex interacting proteins (M247) | 6.4E-01 | 3.0E-03 | 4.8E-03 | 4.5E-01 | 2.7E-01 | 7.9E-03 | 1.6E-01 | 4.9E-01 | 5.7E-01 | 6.2E-02 | 1.7E-02 | 3.1E-01 | 1.0E+00 | 9.9E-01 | 1.0E+00 |
| enriched in plasma membrane proteins (I) (M135.0) | 9.7E-01 | 4.9E-02 | 7.7E-01 | 7.6E-01 | 8.8E-01 | 3.5E-01 | 8.4E-01 | 8.9E-01 | 6.9E-01 | 9.1E-01 | 8.8E-01 | 9.7E-01 | 1.0E+00 | 9.9E-01 | 1.0E+00 |
| enriched in plasma membrane proteins (II) (M135.1) | 9.7E-01 | 4.7E-02 | 7.0E-01 | 6.5E-01 | 7.7E-01 | 5.2E-01 | 4.7E-01 | 8.5E-01 | 4.3E-01 | 7.7E-01 | 5.1E-01 | 2.2E-01 | 1.0E+00 | 9.9E-01 | 1.0E+00 |
| enriched in T cells (I) (M7.0) | 6.4E-01 | 6.8E-04 | 1.0E-03 | 2.0E-01 | 1.4E-04 | 2.6E-01 | 9.1E-01 | 4.6E-01 | 2.4E-02 | 1.8E-07 | 1.0E-09 | 5.2E-11 | 1.0E+00 | 9.9E-01 | 1.0E+00 |
| enriched in T cells (II) (M223) | 9.4E-01 | 1.9E-03 | 5.5E-03 | 4.8E-01 | 3.9E-02 | 4.7E-03 | 1.8E-01 | 8.9E-01 | 6.0E-01 | 8.2E-06 | 2.8E-03 | 1.4E-04 | 1.0E+00 | 9.9E-01 | 1.0E+00 |
| erythrocyte differentiation (M173) | 7.0E-01 | 9.3E-03 | 2.4E-02 | 2.4E-01 | 7.4E-02 | 3.9E-02 | 2.6E-01 | 3.0E-01 | 7.0E-01 | 6.1E-03 | 1.4E-01 | 6.9E-01 | 1.0E+00 | 9.9E-01 | 1.0E+00 |
| extracellular matrix (I) (M2.0) | 7.0E-01 | 8.5E-01 | 8.6E-01 | 3.9E-01 | 9.1E-01 | 9.3E-01 | 8.7E-01 | 8.6E-01 | 7.5E-01 | 2.9E-01 | 3.6E-02 | 3.8E-02 | 1.0E+00 | 9.9E-01 | 1.0E+00 |
| extracellular matrix (II) (M2.1) | 8.0E-01 | 1.5E-03 | 2.4E-02 | 3.7E-01 | 3.7E-01 | 2.3E-03 | 1.1E-01 | 9.8E-01 | 5.9E-01 | 1.5E-07 | 6.2E-06 | 1.7E-04 | 1.0E+00 | 9.9E-01 | 1.0E+00 |
| extracellular matrix, collagen (M210) | 7.0E-01 | 5.6E-03 | 1.3E-02 | 3.1E-01 | 5.4E-03 | 1.9E-02 | 5.0E-01 | 9.9E-01 | 8.8E-01 | 6.3E-02 | 5.5E-02 | 2.5E-02 | 1.0E+00 | 9.9E-01 | 1.0E+00 |
| extracellular matrix, complement (M140) | 8.4E-01 | 4.2E-03 | 1.3E-02 | 5.9E-01 | 3.9E-01 | 1.2E-01 | 3.7E-01 | 9.4E-01 | 5.5E-01 | 3.0E-04 | 5.8E-02 | 2.7E-02 | 1.0E+00 | 9.5E-01 | 1.0E+00 |
| formyl peptide receptor mediated neutrophil response (M11.2) | 8.8E-01 | 6.8E-04 | 1.4E-03 | 3.7E-01 | 1.8E-02 | 9.5E-04 | 5.1E-02 | 9.4E-01 | 6.3E-01 | 2.4E-05 | 3.2E-03 | 7.6E-04 | 1.0E+00 | 9.9E-01 | 1.0E+00 |
| G protein mediated calcium signaling (M159) | 7.1E-01 | 8.1E-01 | 2.5E-01 | 2.4E-01 | 4.6E-01 | 8.4E-01 | 4.7E-01 | 8.9E-01 | 9.4E-01 | 1.1E-02 | 8.9E-03 | 1.0E-02 | 1.0E+00 | 1.0E+00 | 1.0E+00 |
| glycerophospholipid metabolism (M114.1) | 9.4E-01 | 7.9E-02 | 1.2E-01 | 4.7E-01 | 2.5E-01 | 4.6E-01 | 1.6E-01 | 9.2E-01 | 6.7E-01 | 2.8E-01 | 1.1E-02 | 6.3E-01 | 1.0E+00 | 9.9E-01 | 1.0E+00 |
| golgi membrane (I) (M113) | 8.0E-01 | 7.7E-01 | 8.2E-01 | 9.1E-01 | 2.3E-02 | 6.6E-01 | 8.4E-01 | 8.9E-01 | 3.0E-01 | 5.2E-02 | 4.5E-03 | 1.9E-01 | 1.0E+00 | 9.9E-01 | 1.0E+00 |
| golgi membrane (II) (M237) | 9.4E-01 | 9.6E-04 | 5.0E-04 | 6.9E-02 | 2.0E-05 | 3.3E-06 | 3.2E-02 | 7.6E-01 | 6.3E-01 | 2.7E-07 | 2.5E-01 | 1.2E-02 | 1.0E+00 | 8.7E-01 | 1.0E+00 |
| growth factor induced, enriched in nuclear receptor subfamily 4 (M94) | 6.4E-01 | 1.3E-03 | 8.2E-02 | 4.8E-01 | 3.2E-01 | 2.0E-01 | 2.4E-01 | 8.0E-01 | 9.7E-01 | 2.1E-03 | 9.2E-01 | 5.6E-03 | 1.0E+00 | 9.9E-01 | 1.0E+00 |
| heme biosynthesis (I) (M171) | 3.2E-01 | 1.5E-01 | 5.7E-02 | 6.0E-01 | 7.8E-02 | 5.8E-01 | 2.2E-01 | 4.3E-02 | 3.9E-01 | 6.9E-04 | 8.3E-04 | 1.4E-01 | 1.0E+00 | 9.5E-01 | 1.0E+00 |
| heme biosynthesis (II) (M222) | 8.5E-01 | 4.4E-01 | 6.0E-01 | 4.0E-01 | 1.6E-01 | 9.6E-01 | 2.7E-01 | 7.8E-01 | 7.2E-01 | 3.9E-01 | 4.8E-02 | 8.6E-01 | 1.0E+00 | 9.9E-01 | 1.0E+00 |
| Hox cluster I (M17.0) | 7.1E-01 | 5.5E-01 | 9.1E-01 | 7.4E-01 | 9.1E-01 | 3.2E-01 | 4.6E-01 | 8.9E-01 | 9.5E-01 | 4.1E-02 | 2.6E-01 | 2.5E-01 | 1.0E+00 | 9.9E-01 | 1.0E+00 |
| Hox cluster III (M17.2) | 7.1E-01 | 3.7E-01 | 4.0E-02 | 8.6E-01 | 2.9E-01 | 7.2E-02 | 7.4E-01 | 9.5E-01 | 9.4E-01 | 7.6E-01 | 4.7E-01 | 4.4E-01 | 1.0E+00 | 9.9E-01 | 1.0E+00 |
| Hox cluster IV (M17.3) | 9.4E-01 | 5.3E-01 | 2.6E-01 | 9.2E-01 | 8.8E-01 | 5.5E-01 | 7.8E-01 | 8.2E-01 | 9.2E-01 | 7.9E-01 | 1.2E-01 | 2.3E-02 | 1.0E+00 | 9.9E-01 | 1.0E+00 |
| Hox cluster VI (M107) | 9.1E-01 | 4.5E-01 | 3.5E-01 | 9.2E-01 | 9.7E-01 | 2.7E-01 | 5.5E-01 | 7.2E-01 | 9.0E-01 | 9.1E-01 | 1.3E-01 | 1.2E-03 | 1.0E+00 | 9.9E-01 | 1.0E+00 |
| IL2, IL7, TCR network (M65) | 7.0E-01 | 2.4E-01 | 2.1E-01 | 5.2E-01 | 9.2E-01 | 9.7E-01 | 8.0E-01 | 7.9E-01 | 8.3E-01 | 1.2E-01 | 5.1E-04 | 1.5E-04 | 1.0E+00 | 9.9E-01 | 1.0E+00 |
| immune activation - generic cluster (M37.0) | 8.0E-01 | 6.2E-03 | 2.6E-01 | 9.2E-02 | 2.5E-02 | 5.6E-01 | 4.1E-01 | 8.9E-01 | 1.7E-01 | 5.3E-11 | 8.7E-12 | 6.7E-10 | 1.0E+00 | 9.9E-01 | 1.0E+00 |
| immuregulation - monocytes, T and B cells (M57) | 9.4E-01 | 7.7E-03 | 3.7E-01 | 5.7E-01 | 5.5E-01 | 6.0E-01 | 3.9E-01 | 7.8E-01 | 6.3E-01 | 8.7E-02 | 1.7E-01 | 4.3E-01 | 1.0E+00 | 9.9E-01 | 1.0E+00 |
| inflammasome receptors and signaling (M53) | 7.2E-01 | 8.7E-03 | 9.4E-02 | 4.0E-01 | 6.8E-01 | 6.9E-02 | 8.5E-02 | 8.9E-01 | 9.4E-01 | 6.9E-05 | 1.8E-02 | 2.6E-03 | 1.0E+00 | 9.9E-01 | 1.0E+00 |
| inflammatory response (M33) | 7.7E-01 | 1.9E-03 | 5.1E-03 | 3.9E-01 | 2.2E-02 | 4.4E-03 | 2.8E-01 | 8.9E-01 | 5.1E-01 | 5.1E-06 | 1.2E-04 | 2.0E-05 | 1.0E+00 | 9.9E-01 | 1.0E+00 |
| innate activation by cytosolic DNA sensing (M13) | 9.4E-01 | 8.1E-02 | 4.6E-02 | 6.2E-01 | 5.3E-02 | 4.3E-02 | 7.4E-01 | 6.5E-01 | 9.0E-01 | 4.0E-01 | 3.3E-01 | 5.3E-01 | 1.0E+00 | 9.9E-01 | 1.0E+00 |
| innate antiviral response (M150) | 7.0E-01 | 7.4E-01 | 4.9E-02 | 7.4E-01 | 3.7E-01 | 6.8E-02 | 4.9E-01 | 9.3E-01 | 8.3E-01 | 7.9E-01 | 1.4E-01 | 9.5E-01 | 1.0E+00 | 9.9E-01 | 1.0E+00 |
| inositol phosphate metabolism (M129) | 7.1E-01 | 3.6E-01 | 3.8E-02 | 1.4E-01 | 2.0E-02 | 1.1E-03 | 5.5E-01 | 4.0E-01 | 9.8E-01 | 2.2E-02 | 1.8E-01 | 4.1E-02 | 1.0E+00 | 9.9E-01 | 1.0E+00 |
| integrin cell surface interactions (I) (M1.0) | 7.1E-01 | 1.8E-01 | 5.2E-03 | 1.5E-01 | 4.9E-01 | 1.2E-01 | 8.9E-01 | 7.1E-01 | 7.6E-01 | 3.3E-01 | 1.4E-02 | 8.1E-03 | 1.0E+00 | 9.9E-01 | 1.0E+00 |
| integrin mediated leukocyte migration (M39) | 9.9E-01 | 1.9E-03 | 2.6E-03 | 6.3E-01 | 4.2E-04 | 3.0E-04 | 4.7E-02 | 5.6E-01 | 5.7E-01 | 6.3E-03 | 2.3E-02 | 9.3E-01 | 1.0E+00 | 9.9E-01 | 1.0E+00 |
| integrins and cell adhesion (M84) | 9.4E-01 | 4.5E-02 | 7.1E-02 | 4.7E-01 | 4.3E-02 | 1.7E-02 | 3.8E-02 | 8.4E-01 | 9.4E-01 | 2.4E-04 | 9.3E-01 | 1.2E-01 | 1.0E+00 | 9.9E-01 | 1.0E+00 |
| intracellular transport (M147) | 7.1E-01 | 8.3E-03 | 1.6E-03 | 8.4E-02 | 8.9E-03 | 1.2E-05 | 1.2E-01 | 7.8E-01 | 9.4E-01 | 1.1E-06 | 6.6E-01 | 2.0E-04 | 1.0E+00 | 7.6E-01 | 1.0E+00 |
| KLF12 targets network (M32.3) | 9.7E-01 | 1.2E-02 | 1.9E-03 | 6.0E-01 | 4.2E-03 | 2.3E-04 | 1.6E-01 | 1.4E-01 | 7.9E-01 | 1.7E-05 | 7.7E-03 | 1.1E-01 | 1.0E+00 | 9.9E-01 | 1.0E+00 |
| leukocyte activation and migration (M45) | 7.2E-01 | 2.2E-02 | 4.6E-02 | 6.5E-01 | 1.3E-03 | 3.6E-03 | 3.2E-01 | 8.9E-01 | 7.9E-01 | 5.4E-02 | 1.2E-01 | 1.8E-01 | 1.0E+00 | 1.0E+00 | 1.0E+00 |
| leukocyte differentiation (M160) | 6.4E-01 | 9.6E-04 | 5.5E-03 | 2.9E-01 | 1.3E-03 | 2.9E-05 | 7.1E-02 | 5.3E-01 | 7.3E-01 | 8.4E-05 | 7.6E-01 | 1.9E-03 | 1.0E+00 | 9.9E-01 | 1.0E+00 |
| lipid metabolism, endoplasmic reticulum (M92) | 7.1E-01 | 1.3E-02 | 5.4E-01 | 9.1E-01 | 9.1E-01 | 8.8E-01 | 4.5E-01 | 8.9E-01 | 7.9E-01 | 1.1E-01 | 5.9E-02 | 2.1E-01 | 1.0E+00 | 9.9E-01 | 1.0E+00 |
| lymphocyte generic cluster (M60) | 9.7E-01 | 4.9E-01 | 4.0E-01 | 1.0E+00 | 6.9E-01 | 9.5E-02 | 2.4E-01 | 5.1E-01 | 9.4E-01 | 5.3E-02 | 6.1E-03 | 1.9E-02 | 1.0E+00 | 9.9E-01 | 1.0E+00 |
| lysosomal/endosomal proteins (M139) | 8.0E-01 | 8.6E-03 | 3.9E-03 | 9.2E-02 | 4.4E-01 | 2.7E-03 | 1.3E-01 | 6.5E-01 | 2.9E-01 | 1.2E-07 | 1.7E-05 | 7.1E-06 | 1.0E+00 | 9.9E-01 | 1.0E+00 |
| lysosome (M209) | 9.7E-01 | 3.5E-01 | 4.8E-02 | 2.4E-01 | 7.5E-01 | 1.0E-01 | 1.5E-01 | 4.9E-01 | 4.4E-01 | 3.0E-06 | 2.7E-04 | 2.8E-06 | 1.0E+00 | 9.9E-01 | 1.0E+00 |
| MAPK, RAS signaling (M100) | 7.3E-01 | 1.4E-03 | 1.4E-03 | 1.9E-01 | 9.9E-04 | 2.4E-04 | 2.5E-02 | 4.9E-01 | 8.8E-01 | 8.0E-05 | 7.1E-01 | 2.1E-02 | 1.0E+00 | 9.9E-01 | 1.0E+00 |
| Membrane, ER proteins (M134) | 7.5E-01 | 5.5E-01 | 8.6E-01 | 7.6E-01 | 2.2E-01 | 2.6E-02 | 3.8E-01 | 9.0E-01 | 6.6E-01 | 4.7E-01 | 4.4E-01 | 3.6E-01 | 1.0E+00 | 1.0E+00 | 1.0E+00 |
| Memory B cell surface signature (S9) | 9.7E-01 | 9.0E-03 | 2.0E-01 | 8.8E-01 | 1.4E-02 | 1.2E-02 | 3.2E-01 | 8.4E-01 | 9.8E-01 | 5.8E-02 | 8.8E-02 | 6.9E-01 | 1.0E+00 | 9.9E-01 | 1.0E+00 |
| MHC-TLR7-TLR8 cluster (M146) | 9.3E-01 | 1.4E-03 | 2.0E-03 | 5.6E-01 | 1.6E-02 | 2.1E-02 | 5.1E-02 | 3.0E-01 | 9.2E-01 | 4.1E-07 | 7.2E-04 | 7.4E-03 | 1.0E+00 | 9.9E-01 | 1.0E+00 |
| mismatch repair (I) (M22.0) | 9.6E-01 | 6.0E-04 | 2.4E-04 | 5.3E-02 | 2.3E-04 | 1.1E-05 | 4.7E-02 | 3.0E-01 | 6.3E-01 | 1.0E-07 | 7.9E-01 | 3.6E-04 | 1.0E+00 | 9.9E-01 | 1.0E+00 |
| mismatch repair (II) (M22.1) | 8.2E-01 | 1.8E-03 | 7.4E-04 | 2.5E-01 | 6.0E-02 | 2.3E-03 | 2.9E-01 | 4.0E-01 | 8.5E-01 | 1.3E-04 | 9.6E-01 | 4.2E-02 | 1.0E+00 | 9.9E-01 | 1.0E+00 |
| mitochondrial cluster (M235) | 8.0E-01 | 1.9E-03 | 2.8E-03 | 1.2E-01 | 2.5E-04 | 3.3E-04 | 1.0E-01 | 4.9E-01 | 9.5E-01 | 1.8E-06 | 8.4E-03 | 4.7E-05 | 1.0E+00 | 9.9E-01 | 1.0E+00 |
| mitosis (TF motif CCAATNNSNNNGCG) (M169) | 9.7E-01 | 6.8E-04 | 4.5E-04 | 6.3E-02 | 5.1E-05 | 4.3E-06 | 3.2E-02 | 2.4E-01 | 9.5E-01 | 1.2E-06 | 9.9E-01 | 8.1E-04 | 1.0E+00 | 8.4E-01 | 1.0E+00 |
| mitotic cell cycle - DNA replication (M4.4) | 7.5E-01 | 1.5E-01 | 7.1E-01 | 6.6E-01 | 9.7E-01 | 8.5E-01 | 8.8E-01 | 6.3E-01 | 2.8E-01 | 3.5E-03 | 7.3E-02 | 9.4E-02 | 1.0E+00 | 9.9E-01 | 1.0E+00 |
| mitotic cell cycle (M4.7) | 9.6E-01 | 1.0E+00 | 1.2E-01 | 8.4E-01 | 7.6E-01 | 9.4E-01 | 4.1E-01 | 7.7E-01 | 7.0E-01 | 1.2E-01 | 1.2E-01 | 3.4E-02 | 1.0E+00 | 1.0E+00 | 1.0E+00 |
| mitotic cell cycle in stimulated CD4 T cells (M4.11) | 8.8E-01 | 1.1E-02 | 1.0E-01 | 9.6E-01 | 6.9E-01 | 6.6E-01 | 9.1E-01 | 3.7E-01 | 3.6E-01 | 3.1E-02 | 5.1E-01 | 1.9E-02 | 1.0E+00 | 7.2E-01 | 1.0E+00 |
| mitotic cell cycle in stimulated CD4 T cells (M4.5) | 8.1E-01 | 9.6E-04 | 2.4E-02 | 3.9E-01 | 5.2E-02 | 9.3E-01 | 9.1E-01 | 4.9E-01 | 1.7E-01 | 2.7E-04 | 1.5E-02 | 8.3E-04 | 1.0E+00 | 9.9E-01 | 1.0E+00 |
| mitotic cell cycle in stimulated CD4 T cells (M4.9) | 7.0E-01 | 1.7E-02 | 8.5E-02 | 6.2E-01 | 1.6E-01 | 1.2E-01 | 7.0E-01 | 3.4E-01 | 3.7E-01 | 2.9E-02 | 9.9E-01 | 5.0E-01 | 1.0E+00 | 9.9E-01 | 1.0E+00 |
| mitotic cell division (M6) | 9.6E-01 | 6.9E-04 | 2.5E-03 | 8.7E-01 | 4.2E-01 | 1.2E-01 | 3.9E-01 | 1.4E-01 | 1.4E-01 | 1.3E-05 | 4.5E-01 | 2.9E-02 | 1.0E+00 | 9.9E-01 | 1.0E+00 |
| Monocyte surface signature (S4) | 6.4E-01 | 5.8E-05 | 6.3E-05 | 3.7E-02 | 4.3E-01 | 4.3E-06 | 5.0E-03 | 9.9E-01 | 1.1E-02 | 3.4E-12 | 8.7E-12 | 3.5E-11 | 1.0E+00 | 9.9E-01 | 1.0E+00 |
| myeloid cell enriched receptors and transporters (M4.3) | 7.3E-01 | 7.5E-03 | 5.6E-04 | 1.9E-01 | 3.7E-01 | 3.6E-03 | 9.1E-02 | 4.9E-01 | 2.4E-02 | 1.2E-09 | 7.6E-09 | 1.1E-08 | 1.0E+00 | 9.9E-01 | 1.0E+00 |
| myeloid, dendritic cell activation via NFkB (I) (M43.0) | 7.3E-01 | 3.7E-03 | 1.7E-02 | 4.4E-01 | 1.1E-03 | 8.9E-03 | 2.3E-01 | 9.0E-01 | 8.3E-01 | 1.1E-03 | 5.3E-01 | 6.8E-02 | 1.0E+00 | 9.9E-01 | 1.0E+00 |
| myeloid, dendritic cell activation via NFkB (II) (M43.1) | 9.4E-01 | 2.0E-02 | 6.8E-02 | 5.7E-01 | 7.0E-02 | 5.2E-01 | 2.6E-01 | 4.9E-01 | 8.9E-01 | 2.1E-03 | 9.3E-01 | 3.1E-01 | 1.0E+00 | 9.9E-01 | 1.0E+00 |
| NK cell surface signature (S1) | 9.7E-01 | 5.4E-01 | 1.0E-01 | 2.7E-03 | 9.8E-05 | 2.4E-01 | 2.5E-01 | 1.0E+00 | 9.4E-01 | 8.4E-05 | 3.7E-03 | 6.4E-08 | 1.0E+00 | 9.9E-01 | 1.0E+00 |
| nuclear pore complex (M106.0) | 9.4E-01 | 5.7E-03 | 1.3E-02 | 1.2E-01 | 6.8E-05 | 1.1E-05 | 3.4E-02 | 7.9E-01 | 9.4E-01 | 1.2E-06 | 1.7E-02 | 2.7E-05 | 1.0E+00 | 9.9E-01 | 1.0E+00 |
| nuclear pore complex (mitosis) (M106.1) | 9.7E-01 | 1.3E-02 | 9.4E-02 | 2.1E-01 | 4.5E-03 | 1.0E-03 | 7.9E-02 | 8.9E-01 | 9.4E-01 | 2.7E-04 | 4.5E-01 | 1.6E-03 | 1.0E+00 | 9.9E-01 | 1.0E+00 |
| nuclear pore, transport; mRNA splicing, processing (M143) | 9.7E-01 | 9.9E-03 | 7.5E-03 | 1.9E-01 | 3.0E-04 | 3.1E-05 | 4.6E-01 | 1.0E+00 | 9.4E-01 | 6.0E-04 | 8.6E-02 | 9.7E-04 | 1.0E+00 | 9.9E-01 | 1.0E+00 |
| nucleotide metabolism (M181) | 7.3E-01 | 1.4E-03 | 5.5E-03 | 2.7E-01 | 1.1E-03 | 6.9E-04 | 3.2E-01 | 4.0E-01 | 7.0E-01 | 2.4E-05 | 1.8E-02 | 1.1E-03 | 1.0E+00 | 9.9E-01 | 1.0E+00 |
| phosphatidylinositol signaling system (M101) | 6.4E-01 | 1.2E-02 | 1.4E-03 | 3.9E-02 | 4.2E-04 | 1.6E-05 | 1.0E-01 | 4.9E-01 | 9.6E-01 | 4.3E-05 | 4.7E-01 | 6.8E-03 | 1.0E+00 | 7.4E-01 | 1.0E+00 |
| Plasma cell surface signature (S3) | 9.7E-01 | 1.1E-02 | 9.7E-01 | 8.7E-01 | 6.9E-01 | 7.0E-01 | 6.1E-01 | 1.0E+00 | 9.2E-01 | 1.4E-01 | 9.3E-01 | 3.9E-01 | 1.0E+00 | 9.9E-01 | 1.0E+00 |
| plasma cells & B cells, immunoglobulins (M156.0) | 9.7E-01 | 2.1E-03 | 2.1E-01 | 1.3E-03 | 5.1E-04 | 1.7E-03 | 5.2E-01 | 4.9E-01 | 2.8E-01 | 3.3E-03 | 1.1E-07 | 8.5E-04 | 1.0E+00 | 9.9E-01 | 1.0E+00 |
| plasma cells, immunoglobulins (M156.1) | 9.7E-01 | 2.3E-03 | 3.1E-01 | 9.1E-02 | 1.6E-02 | 1.1E-01 | 8.5E-01 | 9.4E-01 | 3.5E-01 | 7.2E-03 | 8.9E-06 | 2.0E-02 | 1.0E+00 | 9.9E-01 | 1.0E+00 |
| plasma membrane, cell junction (M162.0) | 7.0E-01 | 3.2E-02 | 5.8E-03 | 9.8E-01 | 6.3E-01 | 5.3E-01 | 8.6E-01 | 5.5E-01 | 7.7E-01 | 6.5E-02 | 6.0E-01 | 3.1E-01 | 1.0E+00 | 9.9E-01 | 1.0E+00 |
| platelet activation - actin binding (M196) | 7.0E-01 | 6.8E-04 | 8.9E-05 | 4.5E-02 | 3.5E-01 | 1.6E-04 | 7.4E-01 | 9.4E-01 | 7.6E-01 | 3.0E-01 | 2.3E-07 | 2.8E-06 | 1.0E+00 | 9.9E-01 | 1.0E+00 |
| platelet activation & blood coagulation (M199) | 7.0E-01 | 2.4E-03 | 8.5E-04 | 5.6E-02 | 3.0E-01 | 2.2E-02 | 8.8E-01 | 1.0E+00 | 9.5E-01 | 4.1E-01 | 5.7E-04 | 2.3E-03 | 1.0E+00 | 9.6E-01 | 1.0E+00 |
| platelet activation (I) (M32.0) | 9.7E-01 | 1.4E-03 | 4.8E-04 | 1.9E-01 | 9.8E-05 | 5.0E-05 | 3.8E-02 | 3.7E-01 | 9.7E-01 | 7.0E-07 | 3.3E-01 | 1.3E-02 | 1.0E+00 | 9.9E-01 | 1.0E+00 |
| platelet activation (II) (M32.1) | 7.5E-01 | 9.3E-04 | 5.6E-04 | 4.4E-02 | 5.2E-05 | 1.1E-05 | 1.7E-02 | 2.2E-01 | 9.4E-01 | 5.7E-08 | 4.7E-01 | 3.2E-04 | 1.0E+00 | 9.9E-01 | 1.0E+00 |
| platelet activation (III) (M42) | 8.8E-01 | 1.7E-03 | 1.8E-03 | 4.3E-01 | 4.5E-02 | 6.8E-02 | 7.1E-02 | 9.4E-01 | 9.4E-01 | 1.3E-05 | 4.3E-03 | 6.5E-04 | 1.0E+00 | 9.9E-01 | 1.0E+00 |
| platelet activation and degranulation (M85) | 7.0E-01 | 4.9E-01 | 8.7E-01 | 2.1E-01 | 1.5E-01 | 9.2E-01 | 5.3E-01 | 9.8E-01 | 8.3E-01 | 1.3E-02 | 3.5E-04 | 6.5E-04 | 1.0E+00 | 9.9E-01 | 1.0E+00 |
| PLK1 signaling events (M4.2) | 9.4E-01 | 6.8E-04 | 2.7E-02 | 5.6E-01 | 5.9E-01 | 2.6E-01 | 3.3E-01 | 3.4E-01 | 5.1E-01 | 1.2E-04 | 9.6E-01 | 6.6E-02 | 1.0E+00 | 9.9E-01 | 1.0E+00 |
| proinflammatory cytokines and chemokines (M29) | 8.0E-01 | 1.2E-03 | 2.6E-01 | 9.5E-01 | 6.9E-01 | 1.8E-01 | 5.5E-01 | 8.3E-01 | 9.0E-01 | 3.9E-01 | 4.7E-01 | 6.6E-01 | 1.0E+00 | 9.9E-01 | 1.0E+00 |
| proinflammatory dendritic cell, myeloid cell response (M86.1) | 9.1E-01 | 2.2E-02 | 2.8E-01 | 8.8E-01 | 2.6E-01 | 2.6E-01 | 6.7E-01 | 9.9E-01 | 9.2E-01 | 4.2E-01 | 3.3E-01 | 9.4E-02 | 1.0E+00 | 7.6E-01 | 1.0E+00 |
| proteasome (M226) | 7.0E-01 | 6.8E-04 | 5.6E-04 | 6.9E-02 | 1.1E-03 | 6.3E-05 | 7.6E-02 | 1.6E-01 | 9.5E-01 | 4.1E-07 | 1.5E-01 | 2.9E-04 | 1.0E+00 | 6.3E-01 | 1.0E+00 |
| purine nucleotide biosynthesis (M212) | 6.4E-01 | 5.6E-03 | 8.6E-03 | 6.9E-02 | 5.0E-02 | 3.7E-01 | 4.9E-01 | 6.5E-02 | 2.7E-01 | 3.2E-05 | 2.2E-02 | 2.0E-05 | 1.0E+00 | 7.6E-01 | 1.0E+00 |
| putative targets of PAX3 (M89.0) | 6.4E-01 | 7.0E-04 | 4.7E-02 | 4.6E-01 | 2.2E-01 | 1.4E-03 | 1.2E-01 | 9.5E-01 | 6.9E-01 | 1.1E-02 | 7.7E-01 | 7.7E-03 | 1.0E+00 | 9.9E-01 | 1.0E+00 |
| putative targets of PAX3 (M89.1) | 6.4E-01 | 2.1E-03 | 7.4E-02 | 4.1E-01 | 3.1E-02 | 1.6E-03 | 1.2E-01 | 8.0E-01 | 5.1E-01 | 1.0E-02 | 9.7E-01 | 2.3E-02 | 1.0E+00 | 9.9E-01 | 1.0E+00 |
| RA, WNT, CSF receptors network (monocyte) (M23) | 6.4E-01 | 6.8E-04 | 4.6E-02 | 5.9E-01 | 7.0E-01 | 8.0E-02 | 1.2E-01 | 6.2E-01 | 4.1E-01 | 1.4E-04 | 6.3E-01 | 1.0E-03 | 1.0E+00 | 9.9E-01 | 1.0E+00 |
| Ran mediated mitosis (M15) | 6.4E-01 | 6.6E-03 | 2.6E-01 | 8.5E-01 | 8.3E-01 | 9.2E-01 | 6.8E-01 | 4.9E-01 | 7.7E-01 | 4.1E-02 | 2.4E-01 | 4.6E-01 | 1.0E+00 | 9.9E-01 | 1.0E+00 |
| recruitment of neutrophils (M132) | 9.7E-01 | 2.6E-03 | 3.8E-02 | 9.1E-01 | 5.4E-01 | 3.4E-01 | 4.9E-02 | 4.9E-01 | 5.0E-01 | 6.2E-03 | 4.1E-01 | 4.0E-01 | 1.0E+00 | 9.9E-01 | 1.0E+00 |
| regulation of antigen presentation and immune response (M5.0) | 9.7E-01 | 9.6E-04 | 6.2E-03 | 9.7E-01 | 6.3E-01 | 4.6E-01 | 8.7E-02 | 1.0E+00 | 9.9E-01 | 4.1E-07 | 4.7E-02 | 6.5E-04 | 1.0E+00 | 9.9E-01 | 1.0E+00 |
| regulation of localization (GO) (M63) | 7.1E-01 | 5.6E-03 | 1.4E-02 | 1.0E-01 | 4.2E-04 | 3.4E-04 | 1.4E-01 | 7.2E-01 | 9.2E-01 | 3.4E-04 | 9.5E-01 | 2.8E-02 | 1.0E+00 | 9.9E-01 | 1.0E+00 |
| regulation of signal transduction (M3) | 7.0E-01 | 6.7E-03 | 4.3E-02 | 2.9E-01 | 3.0E-01 | 1.7E-02 | 1.2E-01 | 8.1E-01 | 4.3E-01 | 2.7E-07 | 9.2E-08 | 2.4E-07 | 1.0E+00 | 9.9E-01 | 1.0E+00 |
| regulation of transcription, transcription factors (M213) | 5.7E-01 | 4.1E-01 | 2.6E-01 | 6.9E-02 | 9.2E-05 | 3.0E-05 | 5.7E-01 | 7.8E-01 | 5.5E-01 | 4.5E-01 | 7.8E-01 | 6.5E-01 | 1.0E+00 | 9.9E-01 | 1.0E+00 |
| respiratory electron transport chain (mitochondrion) (M216) | 8.3E-01 | 7.0E-04 | 7.4E-04 | 8.7E-01 | 4.8E-01 | 3.6E-01 | 1.7E-02 | 3.7E-01 | 8.1E-01 | 1.8E-04 | 1.1E-02 | 2.0E-02 | 1.0E+00 | 7.4E-01 | 1.0E+00 |
| respiratory electron transport chain (mitochondrion) (M219) | 8.4E-01 | 7.8E-04 | 1.4E-03 | 6.9E-01 | 4.7E-01 | 2.9E-01 | 1.7E-02 | 2.8E-01 | 8.8E-01 | 1.5E-02 | 3.4E-03 | 2.0E-01 | 1.0E+00 | 7.4E-01 | 1.0E+00 |
| respiratory electron transport chain (mitochondrion) (M231) | 7.0E-01 | 1.9E-03 | 4.5E-03 | 7.1E-01 | 8.4E-02 | 8.0E-01 | 4.2E-02 | 3.7E-01 | 8.4E-01 | 9.0E-03 | 3.3E-01 | 8.9E-02 | 1.0E+00 | 7.7E-01 | 1.0E+00 |
| respiratory electron transport chain (mitochondrion) (M238) | 9.1E-01 | 9.6E-04 | 9.2E-03 | 5.9E-01 | 6.1E-01 | 3.9E-01 | 1.5E-02 | 4.0E-01 | 6.7E-01 | 2.8E-04 | 2.5E-01 | 4.7E-02 | 1.0E+00 | 7.6E-01 | 1.0E+00 |
| Resting dendritic cell surface signature (S10) | 6.4E-01 | 1.7E-01 | 3.0E-02 | 3.8E-02 | 2.5E-01 | 4.2E-01 | 6.0E-01 | 7.8E-01 | 1.2E-01 | 9.0E-09 | 7.6E-09 | 7.5E-09 | 1.0E+00 | 9.9E-01 | 1.0E+00 |
| Rho GTPase cycle (M4.14) | 9.9E-01 | 5.6E-03 | 4.2E-01 | 4.1E-01 | 7.9E-01 | 6.3E-01 | 6.4E-01 | 7.1E-01 | 8.1E-01 | 1.8E-01 | 2.7E-01 | 8.8E-01 | 1.0E+00 | 9.9E-01 | 1.0E+00 |
| RIG-1 like receptor signaling (M68) | 8.5E-01 | 1.7E-03 | 1.4E-01 | 9.2E-01 | 8.7E-01 | 1.9E-01 | 4.7E-01 | 7.1E-01 | 9.4E-01 | 1.2E-01 | 3.2E-01 | 5.2E-01 | 1.0E+00 | 9.9E-01 | 1.0E+00 |
| signaling in T cells (I) (M35.0) | 7.0E-01 | 2.4E-03 | 4.5E-02 | 4.0E-01 | 5.5E-01 | 2.4E-02 | 2.5E-01 | 3.7E-01 | 9.5E-01 | 1.7E-01 | 5.2E-04 | 5.4E-01 | 1.0E+00 | 9.9E-01 | 1.0E+00 |
| signaling in T cells (II) (M35.1) | 9.7E-01 | 3.3E-01 | 1.0E-01 | 1.4E-01 | 4.5E-03 | 7.2E-02 | 5.2E-01 | 8.9E-01 | 9.4E-01 | 2.7E-01 | 5.9E-03 | 1.7E-03 | 1.0E+00 | 9.9E-01 | 1.0E+00 |
| small GTPase mediated signal transduction (M215) | 9.4E-01 | 1.9E-03 | 1.4E-03 | 2.1E-01 | 1.7E-02 | 8.4E-04 | 6.5E-02 | 1.4E-01 | 9.7E-01 | 2.9E-05 | 5.2E-04 | 6.9E-01 | 1.0E+00 | 9.9E-01 | 1.0E+00 |
| spliceosome (M250) | 7.1E-01 | 1.9E-03 | 1.4E-03 | 2.9E-01 | 6.3E-01 | 1.7E-01 | 1.4E-01 | 3.7E-01 | 9.4E-01 | 2.0E-03 | 9.7E-01 | 7.1E-03 | 1.0E+00 | 5.9E-01 | 1.0E+00 |
| suppression of MAPK signaling (M56) | 7.0E-01 | 1.9E-03 | 2.3E-02 | 5.9E-01 | 9.0E-01 | 9.1E-02 | 1.2E-01 | 5.5E-01 | 4.1E-01 | 1.6E-03 | 2.3E-01 | 3.1E-03 | 1.0E+00 | 9.9E-01 | 1.0E+00 |
| T & B cell development, activation (M62.0) | 8.5E-01 | 6.1E-01 | 2.1E-01 | 6.8E-01 | 9.7E-01 | 4.1E-02 | 7.1E-01 | 6.6E-01 | 7.6E-01 | 7.4E-02 | 2.3E-01 | 5.5E-02 | 1.0E+00 | 9.9E-01 | 1.0E+00 |
| T cell activation (I) (M7.1) | 6.4E-01 | 1.4E-01 | 2.6E-01 | 9.2E-01 | 1.5E-02 | 5.1E-01 | 1.9E-01 | 8.9E-01 | 1.1E-02 | 3.9E-02 | 5.8E-09 | 4.1E-09 | 1.0E+00 | 9.9E-01 | 1.0E+00 |
| T cell activation (II) (M7.3) | 6.4E-01 | 1.4E-03 | 5.9E-02 | 3.9E-01 | 4.0E-03 | 2.4E-01 | 9.4E-01 | 7.9E-01 | 5.3E-02 | 4.5E-06 | 6.8E-08 | 8.9E-08 | 1.0E+00 | 1.0E+00 | 1.0E+00 |
| T cell activation (III) (M7.4) | 9.7E-01 | 7.9E-01 | 9.1E-02 | 9.4E-01 | 6.3E-01 | 9.3E-01 | 1.2E-01 | 7.8E-01 | 3.3E-02 | 5.2E-01 | 7.7E-03 | 1.6E-02 | 1.0E+00 | 9.9E-01 | 1.0E+00 |
| T cell activation (IV) (M52) | 9.6E-01 | 6.3E-02 | 2.6E-01 | 8.7E-01 | 9.4E-03 | 9.9E-03 | 7.1E-02 | 7.8E-01 | 8.1E-01 | 5.6E-03 | 4.7E-01 | 5.5E-01 | 1.0E+00 | 9.9E-01 | 1.0E+00 |
| T cell activation and signaling (M5.1) | 7.0E-01 | 8.5E-01 | 8.3E-01 | 6.5E-01 | 2.7E-02 | 1.2E-01 | 3.6E-01 | 6.1E-01 | 3.9E-01 | 4.6E-01 | 6.1E-03 | 3.9E-04 | 1.0E+00 | 9.9E-01 | 1.0E+00 |
| T cell differentiation (M14) | 9.7E-01 | 2.2E-01 | 4.1E-01 | 6.0E-01 | 7.7E-02 | 6.3E-01 | 5.4E-02 | 8.3E-01 | 1.7E-01 | 7.3E-01 | 7.7E-03 | 2.7E-02 | 1.0E+00 | 9.9E-01 | 1.0E+00 |
| T cell differentiation (Th2) (M19) | 6.4E-01 | 8.3E-03 | 6.2E-04 | 6.5E-01 | 5.8E-02 | 6.7E-01 | 9.1E-01 | 3.7E-01 | 2.2E-01 | 2.4E-04 | 2.5E-02 | 1.7E-05 | 1.0E+00 | 9.9E-01 | 1.0E+00 |
| T cell differentiation via ITK and PKC (M18) | 7.3E-01 | 3.6E-01 | 2.2E-02 | 9.6E-01 | 7.9E-02 | 9.3E-01 | 4.7E-01 | 8.4E-01 | 3.3E-01 | 9.6E-02 | 3.2E-03 | 1.7E-04 | 1.0E+00 | 9.9E-01 | 1.0E+00 |
| T cell signaling and costimulation (M44) | 9.7E-01 | 8.7E-03 | 2.4E-02 | 9.1E-01 | 1.9E-03 | 4.8E-03 | 4.2E-02 | 8.4E-01 | 6.0E-01 | 2.7E-03 | 9.1E-01 | 4.7E-01 | 1.0E+00 | 9.9E-01 | 1.0E+00 |
| T cell surface signature (S0) | 6.4E-01 | 9.6E-04 | 5.6E-04 | 5.5E-01 | 5.6E-01 | 9.2E-01 | 6.4E-01 | 3.7E-01 | 2.2E-01 | 1.4E-06 | 2.4E-05 | 5.5E-07 | 1.0E+00 | 9.9E-01 | 1.0E+00 |
| T cell surface, activation (M36) | 9.7E-01 | 5.6E-03 | 2.9E-02 | 6.8E-01 | 3.9E-04 | 1.6E-03 | 8.6E-02 | 7.8E-01 | 7.5E-01 | 3.3E-03 | 6.9E-01 | 2.7E-01 | 1.0E+00 | 9.9E-01 | 1.0E+00 |
| targets of FOSL1/2 (M0) | 7.2E-01 | 1.4E-03 | 4.4E-01 | 7.5E-01 | 9.4E-01 | 9.1E-01 | 4.1E-01 | 8.9E-01 | 9.5E-01 | 5.5E-01 | 3.0E-01 | 5.3E-01 | 1.0E+00 | 9.9E-01 | 1.0E+00 |
| TLR and inflammatory signaling (M16) | 7.0E-01 | 1.1E-04 | 2.2E-04 | 7.8E-02 | 4.8E-01 | 7.4E-04 | 5.2E-02 | 4.9E-01 | 3.3E-02 | 1.8E-09 | 2.1E-08 | 4.3E-08 | 1.0E+00 | 9.9E-01 | 1.0E+00 |
| TLR8-BAFF network (M25) | 7.0E-01 | 7.0E-02 | 5.7E-01 | 3.9E-01 | 5.6E-01 | 3.4E-01 | 4.9E-01 | 7.1E-01 | 5.2E-01 | 2.4E-04 | 2.4E-05 | 2.7E-04 | 1.0E+00 | 9.9E-01 | 1.0E+00 |
| transcription elongation, RNA polymerase II (M234) | 7.0E-01 | 8.3E-03 | 8.2E-01 | 4.7E-01 | 1.1E-01 | 1.1E-03 | 1.7E-02 | 4.9E-01 | 8.3E-01 | 1.2E-04 | 2.6E-01 | 2.4E-02 | 1.0E+00 | 7.6E-01 | 1.0E+00 |
| transcription regulation in cell development (M49) | 7.5E-01 | 2.4E-01 | 3.8E-02 | 1.4E-01 | 5.9E-01 | 1.4E-02 | 4.6E-01 | 7.2E-01 | 5.1E-01 | 8.4E-05 | 1.5E-03 | 1.0E-05 | 1.0E+00 | 9.9E-01 | 1.0E+00 |
| transcriptional targets of glucocorticoid receptor (M74) | 7.1E-01 | 4.9E-02 | 5.9E-02 | 3.2E-01 | 6.8E-02 | 2.1E-02 | 2.9E-01 | 8.2E-01 | 7.7E-01 | 9.0E-01 | 5.8E-02 | 4.5E-01 | 1.0E+00 | 1.0E+00 | 1.0E+00 |
| translation initiation (M227) | 9.4E-01 | 1.5E-03 | 1.7E-03 | 4.0E-01 | 1.6E-02 | 1.1E-02 | 1.6E-01 | 3.1E-01 | 8.8E-01 | 2.5E-04 | 7.2E-01 | 5.1E-03 | 1.0E+00 | 5.9E-01 | 1.0E+00 |
| translation initiation factor 3 complex (M245) | 7.0E-01 | 1.2E-02 | 3.4E-02 | 4.5E-02 | 1.6E-02 | 1.6E-01 | 1.3E-01 | 3.7E-01 | 7.7E-01 | 1.5E-02 | 1.6E-02 | 2.4E-03 | 1.0E+00 | 9.9E-01 | 1.0E+00 |
| transmembrane transport (I) (M87) | 7.1E-01 | 1.1E-03 | 4.2E-03 | 2.0E-01 | 2.7E-01 | 1.6E-03 | 3.2E-02 | 9.5E-01 | 5.1E-01 | 3.6E-07 | 6.8E-04 | 1.9E-05 | 1.0E+00 | 9.9E-01 | 1.0E+00 |
| transmembrane transport (II) (M191) | 7.1E-01 | 2.5E-01 | 1.0E-02 | 3.6E-01 | 2.3E-02 | 2.2E-04 | 3.8E-01 | 7.6E-01 | 8.8E-01 | 2.5E-02 | 7.0E-01 | 2.1E-02 | 1.0E+00 | 9.9E-01 | 1.0E+00 |
| type I interferon response (M127) | 6.4E-01 | 1.6E-01 | 7.3E-01 | 6.5E-01 | 4.3E-01 | 7.7E-01 | 8.6E-01 | 9.5E-01 | 9.8E-01 | 4.6E-01 | 2.4E-01 | 1.5E-02 | 1.0E+00 | 9.9E-01 | 1.0E+00 |
| viral sensing & immunity; IRF2 targets network (I) (M111.0) | 9.9E-01 | 4.8E-01 | 5.7E-02 | 3.9E-01 | 3.9E-01 | 2.6E-02 | 5.2E-01 | 4.6E-01 | 6.6E-01 | 4.6E-05 | 2.2E-05 | 7.4E-04 | 1.0E+00 | 9.9E-01 | 1.0E+00 |
| Wnt signaling pathway (M206) | 7.3E-01 | 4.9E-01 | 3.7E-01 | 8.7E-01 | 1.2E-02 | 1.5E-01 | 4.7E-01 | 8.9E-01 | 5.7E-02 | 7.5E-01 | 1.9E-01 | 9.3E-01 | 1.0E+00 | 9.9E-01 | 1.0E+00 |

1. Li S, Rouphael N, Duraisingham S, Romero-Steiner S, Presnell S, Davis C, Schmidt DS, Johnson SE, Milton A, Rajam G *et al*: **Molecular signatures of antibody responses derived from a systems biology study of five human vaccines**. *Nature immunology* 2014, **15**(2):195-204.

2. Hochberg Y, Benjamini Y: **More powerful procedures for multiple significance testing**. *Statistics in medicine* 1990, **9**(7):811-818.
