# Supplementary material for: Aging-dependent alterations in gene expression and a mitochondrial signature of responsiveness to human influenza vaccination
Source: Aging (Albany NY). 2015 Jan 14;7(1):38–51. doi: 10.18632/aging.100720 (PMC4356402; doi:10.18632/aging.100720)
Supplement: Supplementary file 5 [file aging-07-38-s005.doc]

Supplementary table 4A: Pathway activities obtained by QuSAGE for immunological pathways from the KEGG database by comparing pre- and post-vaccination transcriptional profiles. Note that this table includes all pathways, while Figure 4 includes a subset of these pathways with FDR<0.005.

|  | Young | | | | | | Older | | | | | | Frail | | |
| --- | --- | --- | --- | --- | --- | --- | --- | --- | --- | --- | --- | --- | --- | --- | --- |
|  | R+ | R+ | R+ | R- | R- | R- | R+ | R+ | R+ | R- | R- | R- | R- | R- | R- |
| *Days-post vaccination* | 2 | 7 | 28 | 2 | 7 | 28 | 2 | 7 | 28 | 2 | 7 | 28 | 2 | 7 | 28 |
| ANTIGEN_PROCESSING_AND_PRESENTATION | 2.6E-02 | -1.5E-02 | -7.2E-02 | -1.3E-02 | -3.5E-02 | -4.0E-02 | -5.7E-02 | -8.3E-03 | -5.7E-03 | -4.9E-02 | -4.9E-02 | -2.3E-02 | -8.8E-03 | -4.6E-02 | 1.2E-02 |
| APOPTOSIS | 1.2E-02 | -2.5E-02 | -8.1E-03 | -1.4E-02 | -2.1E-02 | -1.6E-02 | -1.5E-02 | 2.0E-02 | 2.0E-03 | -2.6E-02 | 3.9E-02 | 2.6E-02 | -2.3E-03 | -7.1E-03 | -1.6E-02 |
| B_CELL_RECEPTOR_SIGNALING | -1.7E-02 | -7.2E-02 | -6.9E-02 | -3.5E-03 | -6.9E-03 | -7.0E-03 | -6.4E-02 | 3.0E-02 | -1.9E-02 | -5.5E-02 | 6.2E-02 | 1.7E-02 | -3.9E-02 | -4.2E-04 | -6.6E-03 |
| BASAL_TRANSCRIPTION_FACTORS | 2.0E-02 | 2.5E-02 | 3.6E-02 | 2.8E-02 | 6.1E-03 | 2.9E-02 | 2.7E-02 | 2.0E-02 | 5.6E-02 | 2.0E-02 | -1.6E-03 | 1.3E-02 | 2.9E-03 | -3.4E-02 | 5.3E-03 |
| BASE_EXCISION_REPAIR | 4.0E-02 | 4.7E-02 | 3.2E-02 | 2.3E-02 | 2.4E-02 | 4.8E-03 | 3.5E-02 | 2.7E-02 | 2.4E-02 | 2.2E-02 | -1.7E-02 | -1.8E-02 | 3.1E-04 | 2.3E-02 | 9.5E-03 |
| CELL_ADHESION_MOLECULES_CAMS | 1.2E-03 | -2.4E-02 | -3.2E-02 | -1.0E-02 | -2.3E-02 | -1.0E-02 | -4.6E-02 | -2.3E-02 | -2.9E-02 | -3.5E-02 | -1.2E-02 | -1.7E-02 | 1.8E-03 | -1.3E-02 | 9.1E-03 |
| CELL_CYCLE | -4.6E-03 | 4.9E-02 | 4.8E-02 | 2.5E-03 | 1.9E-02 | 4.1E-02 | 7.9E-03 | 4.3E-03 | 1.0E-02 | -1.1E-02 | -1.2E-02 | -2.7E-02 | -1.2E-02 | -1.8E-02 | -7.2E-03 |
| CHEMOKINE_SIGNALING | -3.3E-02 | -8.9E-02 | -6.8E-02 | -3.0E-02 | -4.1E-02 | -3.1E-02 | -7.1E-02 | -2.5E-02 | -4.4E-02 | -7.5E-02 | 2.7E-02 | 5.4E-04 | -6.8E-03 | -1.6E-03 | -1.6E-02 |
| CITRATE_CYCLE_TCA_CYCLE | 4.0E-02 | 1.1E-01 | 6.8E-02 | 1.4E-02 | 2.0E-02 | 1.6E-02 | 1.7E-02 | -2.9E-02 | 3.2E-02 | 3.2E-02 | -7.4E-02 | -3.3E-03 | -5.7E-03 | -8.8E-02 | -4.7E-03 |
| COMPLEMENT_AND_COAGULATION_CASCADES | -2.2E-02 | -2.6E-02 | -1.0E-02 | -2.2E-02 | -1.8E-02 | -3.8E-03 | -4.6E-02 | -6.3E-02 | -5.6E-02 | -1.4E-02 | -5.3E-03 | 2.4E-02 | 1.0E-02 | -1.9E-02 | -1.6E-02 |
| CYTOKINE_CYTOKINE_RECEPTOR_INTERACTION | -1.7E-02 | -4.3E-02 | -4.0E-02 | -2.2E-02 | -1.8E-02 | -1.6E-02 | -2.4E-02 | -1.2E-04 | -9.8E-03 | -1.9E-02 | 1.9E-02 | 1.4E-02 | -4.0E-04 | 7.4E-03 | -1.8E-03 |
| CYTOSOLIC_DNA_SENSING | 2.4E-03 | -5.0E-02 | -8.1E-02 | -2.5E-02 | -3.1E-02 | -4.7E-02 | -1.0E-02 | 2.7E-02 | 1.5E-02 | -2.4E-03 | 1.9E-02 | 2.9E-03 | -3.6E-03 | 9.1E-03 | 8.5E-03 |
| DNA_REPLICATION | 1.9E-02 | 8.1E-02 | 8.4E-02 | 1.9E-02 | 2.0E-02 | 3.0E-02 | 6.8E-02 | 2.3E-02 | 5.1E-02 | 4.0E-02 | -4.7E-02 | -5.5E-02 | 1.9E-03 | -1.3E-02 | 7.2E-03 |
| ENDOCYTOSIS | -1.4E-02 | -1.3E-02 | -1.4E-02 | -2.6E-02 | -1.1E-02 | 1.6E-03 | -5.0E-02 | -1.2E-02 | -1.0E-02 | -2.5E-02 | 1.9E-02 | 2.1E-02 | 4.4E-03 | 7.7E-04 | -3.1E-03 |
| ERBB_SIGNALING | -2.4E-02 | -5.5E-02 | -4.1E-02 | -2.1E-02 | -2.3E-02 | -1.1E-02 | -6.3E-02 | 1.0E-02 | -2.8E-02 | -6.1E-02 | 3.3E-02 | 1.8E-03 | -1.9E-02 | 7.1E-03 | -5.9E-03 |
| FC_EPSILON_RI_SIGNALING | -1.4E-02 | -9.5E-02 | -1.1E-01 | -3.4E-02 | -6.4E-02 | -5.7E-02 | -7.5E-02 | -1.3E-02 | -4.3E-02 | -7.9E-02 | 1.5E-02 | 2.7E-03 | -1.1E-02 | -4.7E-03 | -4.4E-03 |
| FC_GAMMA_R_MEDIATED_PHAGOCYTOSIS | 1.1E-04 | -6.3E-02 | -6.6E-02 | -2.5E-02 | -3.9E-02 | -2.0E-02 | -8.9E-02 | -2.7E-02 | -5.4E-02 | -7.8E-02 | 2.5E-02 | 3.1E-02 | 6.6E-03 | -1.1E-02 | -1.7E-02 |
| FOCAL_ADHESION | -1.5E-02 | 1.0E-02 | 2.6E-02 | -2.4E-02 | -8.4E-03 | 2.2E-02 | -3.6E-02 | -2.4E-02 | -3.4E-02 | -2.5E-02 | 2.1E-02 | 1.0E-02 | -6.1E-04 | 9.0E-03 | -9.6E-03 |
| GLYCOLYSIS_GLUCONEOGENESIS | 9.1E-04 | -6.7E-02 | -8.4E-02 | -1.8E-02 | -6.4E-02 | -7.6E-02 | -8.4E-02 | -3.1E-02 | -4.2E-02 | -6.8E-02 | -2.2E-02 | -5.7E-03 | 1.6E-03 | -1.3E-02 | 8.8E-03 |
| HEMATOPOIETIC_CELL_LINEAGE | -2.6E-02 | -5.9E-02 | -4.7E-02 | -3.3E-02 | -3.1E-02 | -3.8E-02 | -1.1E-01 | -4.7E-02 | -8.3E-02 | -7.8E-02 | 4.2E-03 | -6.6E-03 | -1.6E-03 | -3.4E-02 | -4.4E-03 |
| INTESTINAL_IMMUNE_NETWORK_FOR_IGA_PRODUCTION | 3.6E-03 | -4.1E-02 | -1.1E-01 | 3.1E-02 | 5.6E-03 | -9.1E-03 | -7.1E-02 | -2.6E-02 | -5.5E-02 | -5.2E-02 | -4.9E-02 | -3.0E-02 | 7.4E-03 | -2.5E-02 | 3.0E-02 |
| JAK_STAT_SIGNALING | 1.7E-03 | -4.8E-02 | -4.0E-02 | -8.3E-03 | -1.4E-02 | -9.6E-03 | -3.3E-02 | 1.8E-02 | -1.6E-02 | -3.6E-02 | 4.1E-02 | 1.1E-02 | 6.1E-03 | 2.2E-02 | -1.0E-02 |
| LYSOSOME | -1.9E-02 | -5.0E-02 | -9.1E-02 | -5.1E-02 | -1.1E-02 | -7.1E-02 | -1.2E-01 | -6.0E-02 | -8.9E-02 | -6.2E-02 | 3.1E-03 | 4.7E-02 | 1.6E-02 | -1.1E-02 | -1.4E-03 |
| MAPK_SIGNALING | -1.5E-02 | -3.7E-02 | -3.2E-02 | -1.5E-02 | -7.8E-03 | -1.6E-02 | -4.1E-02 | -4.1E-04 | -2.7E-02 | -3.9E-02 | 3.0E-02 | 1.2E-02 | -3.8E-03 | 1.4E-02 | -1.1E-02 |
| MISMATCH_REPAIR | 1.3E-02 | 1.2E-01 | 1.4E-01 | 2.3E-02 | 4.6E-02 | 7.0E-02 | 8.2E-02 | 1.1E-02 | 4.3E-02 | 5.4E-02 | -4.6E-02 | -3.6E-02 | 2.6E-02 | -1.7E-02 | 4.3E-03 |
| MTOR_SIGNALING | -1.2E-02 | -5.0E-02 | -4.6E-02 | -1.2E-02 | -2.0E-02 | -2.0E-02 | -3.6E-02 | 1.9E-02 | 1.8E-03 | -4.5E-02 | 2.4E-02 | 2.5E-03 | -2.1E-02 | 2.2E-02 | -1.4E-02 |
| NATURAL_KILLER_CELL_MEDIATED_CYTOTOXICITY | 1.2E-02 | -1.8E-02 | -4.3E-02 | -2.6E-02 | -4.6E-02 | -3.5E-02 | -2.7E-03 | 1.2E-02 | 2.8E-02 | -1.2E-02 | 3.6E-03 | -5.9E-03 | -1.6E-02 | -3.9E-02 | -1.7E-02 |
| NOD_LIKE_RECEPTOR_SIGNALING | -4.0E-02 | -1.2E-01 | -1.0E-01 | -3.1E-02 | -3.3E-02 | -6.4E-02 | -7.6E-02 | -1.0E-02 | -4.7E-02 | -9.6E-02 | 7.8E-03 | -9.7E-03 | -1.6E-02 | 1.4E-03 | -3.8E-03 |
| NUCLEOTIDE_EXCISION_REPAIR | 8.9E-03 | 6.4E-02 | 7.1E-02 | 1.3E-02 | 4.5E-02 | 4.5E-02 | 4.6E-02 | 4.2E-03 | 2.6E-02 | 3.8E-02 | -4.1E-02 | -1.8E-02 | 3.1E-02 | 5.5E-03 | 2.5E-02 |
| OXIDATIVE_PHOSPHORYLATION | 1.8E-02 | 1.2E-01 | 8.3E-02 | -7.2E-03 | 2.2E-02 | -7.4E-03 | 4.8E-02 | -2.1E-02 | 2.9E-02 | 1.2E-01 | -4.6E-02 | 3.6E-02 | -3.7E-02 | -1.1E-01 | -3.3E-03 |
| PHOSPHATIDYLINOSITOL_SIGNALING_SYSTEM | -2.7E-02 | -5.8E-02 | -5.1E-02 | -1.0E-02 | -2.8E-02 | -1.3E-02 | -4.4E-02 | 1.4E-02 | -2.1E-02 | -5.9E-02 | 3.7E-02 | -6.9E-03 | -1.6E-02 | 1.8E-02 | -1.7E-02 |
| PPAR_SIGNALING | -2.2E-02 | -1.2E-02 | -8.5E-03 | -1.6E-03 | 8.2E-03 | 8.8E-03 | -2.9E-02 | -2.5E-02 | -1.5E-02 | 2.4E-04 | 5.8E-03 | 2.8E-02 | -1.6E-03 | -3.7E-02 | -5.6E-03 |
| PROTEASOME | 6.5E-02 | 1.3E-01 | 8.4E-02 | 1.3E-02 | 4.7E-02 | 4.5E-03 | 7.5E-02 | -5.6E-03 | 5.9E-02 | 7.8E-02 | -6.9E-02 | 3.4E-03 | -2.6E-03 | -7.8E-02 | -1.1E-02 |
| PROTEIN_EXPORT | 1.4E-02 | 2.6E-01 | 1.7E-01 | 4.3E-02 | 5.7E-02 | 6.3E-02 | 5.6E-02 | 2.8E-03 | 4.8E-02 | 3.9E-02 | -4.1E-02 | 1.5E-02 | -6.8E-02 | -1.3E-01 | -4.3E-02 |
| REGULATION_OF_AUTOPHAGY | -7.8E-03 | 1.8E-03 | 1.7E-02 | -9.7E-03 | 1.7E-02 | 1.5E-02 | 1.3E-03 | 2.7E-03 | 5.1E-03 | 7.4E-03 | 4.4E-03 | 2.1E-02 | 1.5E-02 | -4.3E-03 | 7.8E-03 |
| RIG_I_LIKE_RECEPTOR_SIGNALING | -5.9E-03 | -5.8E-02 | -4.3E-02 | 1.9E-03 | -2.2E-03 | -1.8E-02 | -1.9E-02 | 2.5E-02 | -3.3E-03 | -2.5E-02 | 2.6E-02 | -9.6E-03 | -1.7E-02 | 4.6E-03 | -4.9E-03 |
| RNA_DEGRADATION | 7.8E-03 | 7.7E-02 | 7.0E-02 | 2.4E-02 | 8.0E-02 | 7.6E-02 | 5.0E-02 | 1.5E-02 | 3.7E-02 | 5.8E-02 | -2.1E-02 | -5.6E-03 | 8.1E-03 | -1.6E-02 | 2.1E-02 |
| RNA_POLYMERASE | 4.0E-02 | 3.4E-02 | -8.9E-04 | 2.6E-02 | 5.9E-03 | -4.5E-03 | 5.2E-02 | 4.1E-02 | 4.4E-02 | 4.9E-02 | -1.9E-02 | -1.6E-03 | -3.8E-02 | -4.8E-02 | 5.3E-03 |
| SPLICEOSOME | 3.3E-02 | 3.4E-02 | 3.8E-02 | 2.6E-02 | 1.5E-02 | 1.8E-02 | -2.2E-03 | 2.4E-02 | 9.9E-03 | -2.2E-02 | -6.8E-03 | -2.4E-02 | -1.6E-02 | -2.9E-02 | -1.3E-02 |
| T_CELL_RECEPTOR_SIGNALING | -5.3E-03 | -5.6E-02 | -3.6E-02 | -1.3E-02 | -4.3E-02 | -4.2E-02 | -3.1E-02 | 3.7E-02 | 8.3E-03 | -6.5E-02 | 2.6E-02 | -2.7E-02 | -2.0E-02 | 2.1E-02 | -6.0E-04 |
| TGF_BETA_SIGNALING | -1.7E-02 | -1.9E-02 | -8.8E-03 | -1.8E-03 | -1.0E-02 | 1.4E-02 | -1.7E-02 | 3.3E-04 | -3.3E-03 | -2.6E-02 | 7.4E-03 | 9.2E-04 | -1.1E-02 | -9.0E-03 | -1.0E-02 |
| TOLL_LIKE_RECEPTOR_SIGNALING | -3.9E-02 | -9.0E-02 | -7.8E-02 | -2.2E-02 | -8.8E-03 | -3.2E-02 | -6.7E-02 | 3.3E-03 | -2.7E-02 | -5.5E-02 | 3.4E-02 | 3.7E-02 | -1.1E-02 | -7.7E-03 | -9.6E-03 |
| UBIQUITIN_MEDIATED_PROTEOLYSIS | -6.0E-03 | 3.0E-02 | 3.7E-02 | 9.7E-03 | 3.4E-02 | 5.0E-02 | 1.3E-02 | 1.4E-02 | 2.1E-02 | 1.2E-02 | 7.8E-03 | 5.3E-03 | -1.5E-03 | -1.3E-02 | -1.1E-03 |
| VEGF_SIGNALING | -2.0E-02 | -4.1E-02 | -3.1E-02 | -2.2E-02 | -3.0E-02 | -2.1E-02 | -3.6E-02 | -6.6E-03 | -1.9E-02 | -5.0E-02 | 1.4E-03 | -2.3E-03 | -1.2E-02 | 1.2E-02 | -6.9E-03 |
