# Supplementary material for: Aging-dependent alterations in gene expression and a mitochondrial signature of responsiveness to human influenza vaccination
Source: Aging (Albany NY). 2015 Jan 14;7(1):38–51. doi: 10.18632/aging.100720 (PMC4356402; doi:10.18632/aging.100720)
Supplement: Supplementary file 6 [file aging-07-38-s006.doc]

Supplementary table 4B: False Discovery Rates (FDRs) associated with vaccination-induced pathway activities. QuSAGE was used to obtain P values for detecting the activity of immunological pathways from the KEGG database by comparing pre- and post-vaccination transcriptional profiles. FDRs were obtained using the method of Benjamini and Hochberg . Note that this table includes all pathways, while Figure 4 includes a subset of these pathways with FDR<0.005.

|  | Young | | | | | | Older | | | | | | Frail | | |
| --- | --- | --- | --- | --- | --- | --- | --- | --- | --- | --- | --- | --- | --- | --- | --- |
|  | R+ | R+ | R+ | R- | R- | R- | R+ | R+ | R+ | R- | R- | R- | R- | R- | R- |
| *Days-post vaccination* | 2 | 7 | 28 | 2 | 7 | 28 | 2 | 7 | 28 | 2 | 7 | 28 | 2 | 7 | 28 |
| ANTIGEN_PROCESSING_AND_PRESENTATION | 7.3E-01 | 4.1E-01 | 6.8E-03 | 4.9E-01 | 2.1E-02 | 1.5E-02 | 8.1E-02 | 1.4E-01 | 9.8E-01 | 1.9E-06 | 5.3E-01 | 7.2E-01 | 1.0E+00 | 9.5E-01 | 9.8E-01 |
| APOPTOSIS | 8.9E-01 | 1.0E-01 | 5.7E-01 | 4.5E-01 | 1.5E-01 | 3.3E-01 | 2.4E-01 | 2.7E-01 | 9.8E-01 | 1.9E-01 | 6.9E-02 | 8.7E-01 | 1.0E+00 | 9.5E-01 | 9.8E-01 |
| B_CELL_RECEPTOR_SIGNALING | 8.2E-01 | 1.1E-03 | 1.3E-02 | 8.7E-01 | 7.3E-01 | 7.7E-01 | 6.6E-02 | 6.7E-02 | 9.8E-01 | 1.2E-05 | 2.8E-02 | 1.6E-01 | 1.0E+00 | 9.5E-01 | 9.8E-01 |
| BASAL_TRANSCRIPTION_FACTORS | 7.4E-01 | 2.1E-01 | 1.1E-01 | 1.7E-01 | 7.3E-01 | 5.5E-02 | 5.0E-01 | 8.8E-01 | 9.8E-01 | 2.5E-02 | 5.0E-02 | 1.7E-04 | 1.0E+00 | 9.5E-01 | 9.8E-01 |
| BASE_EXCISION_REPAIR | 6.3E-01 | 2.5E-02 | 1.5E-01 | 3.0E-01 | 2.2E-01 | 8.3E-01 | 3.4E-01 | 6.8E-01 | 9.8E-01 | 7.8E-03 | 5.0E-02 | 1.1E-01 | 1.0E+00 | 9.5E-01 | 9.8E-01 |
| CELL_ADHESION_MOLECULES_CAMS | 9.5E-01 | 5.9E-02 | 4.3E-02 | 4.3E-01 | 2.1E-02 | 3.7E-01 | 6.9E-02 | 5.2E-01 | 9.8E-01 | 1.4E-07 | 6.5E-03 | 1.7E-03 | 1.0E+00 | 9.5E-01 | 9.8E-01 |
| CELL_CYCLE | 8.9E-01 | 5.5E-03 | 2.0E-02 | 8.7E-01 | 7.7E-02 | 2.4E-04 | 5.0E-01 | 5.2E-01 | 9.8E-01 | 3.6E-01 | 6.6E-01 | 2.5E-01 | 1.0E+00 | 9.5E-01 | 9.8E-01 |
| CHEMOKINE_SIGNALING | 7.0E-01 | 8.7E-06 | 1.5E-03 | 8.4E-02 | 1.2E-03 | 1.9E-02 | 2.0E-02 | 5.2E-01 | 9.8E-01 | 1.0E-08 | 6.5E-03 | 3.0E-05 | 1.0E+00 | 9.5E-01 | 9.8E-01 |
| CITRATE_CYCLE_TCA_CYCLE | 7.0E-01 | 9.1E-04 | 2.0E-02 | 5.8E-01 | 3.4E-01 | 4.7E-01 | 4.2E-01 | 6.7E-02 | 9.8E-01 | 2.5E-01 | 5.0E-02 | 4.4E-02 | 1.0E+00 | 9.5E-01 | 9.8E-01 |
| COMPLEMENT_AND_COAGULATION_CASCADES | 7.5E-01 | 6.1E-02 | 6.0E-01 | 3.0E-01 | 1.5E-01 | 8.4E-01 | 6.5E-01 | 8.8E-01 | 9.8E-01 | 9.4E-04 | 2.0E-05 | 1.1E-04 | 1.0E+00 | 9.5E-01 | 9.8E-01 |
| CYTOKINE_CYTOKINE_RECEPTOR_INTERACTION | 7.5E-01 | 3.4E-03 | 3.7E-02 | 8.4E-02 | 5.3E-02 | 1.4E-01 | 4.6E-01 | 5.2E-01 | 9.8E-01 | 3.9E-04 | 9.9E-01 | 1.7E-01 | 1.0E+00 | 9.5E-01 | 9.8E-01 |
| CYTOSOLIC_DNA_SENSING | 9.5E-01 | 1.6E-02 | 1.7E-03 | 3.0E-01 | 9.0E-02 | 1.5E-02 | 9.9E-01 | 5.2E-01 | 9.8E-01 | 4.3E-01 | 1.9E-02 | 3.1E-01 | 1.0E+00 | 9.5E-01 | 9.8E-01 |
| DNA_REPLICATION | 7.5E-01 | 2.6E-03 | 2.1E-03 | 4.5E-01 | 3.1E-01 | 1.4E-01 | 2.7E-01 | 1.4E-01 | 6.8E-01 | 3.8E-06 | 6.7E-02 | 5.5E-04 | 1.0E+00 | 9.5E-01 | 9.8E-01 |
| ENDOCYTOSIS | 7.5E-01 | 2.7E-01 | 3.0E-01 | 3.7E-02 | 2.3E-01 | 8.9E-01 | 2.0E-01 | 5.2E-01 | 9.8E-01 | 4.7E-10 | 6.7E-02 | 1.6E-01 | 1.0E+00 | 9.5E-01 | 9.8E-01 |
| ERBB_SIGNALING | 7.3E-01 | 2.5E-03 | 4.9E-02 | 3.0E-01 | 1.2E-01 | 4.5E-01 | 2.0E-02 | 4.3E-01 | 9.8E-01 | 1.6E-07 | 4.4E-01 | 1.4E-02 | 1.0E+00 | 9.5E-01 | 9.8E-01 |
| FC_EPSILON_RI_SIGNALING | 7.5E-01 | 3.6E-05 | 8.6E-05 | 8.4E-02 | 2.8E-05 | 8.9E-05 | 2.5E-03 | 6.8E-01 | 9.8E-01 | 1.3E-08 | 2.1E-01 | 6.6E-04 | 1.0E+00 | 9.5E-01 | 9.8E-01 |
| FC_GAMMA_R_MEDIATED_PHAGOCYTOSIS | 9.5E-01 | 7.3E-04 | 2.6E-03 | 2.4E-01 | 6.7E-03 | 2.1E-01 | 1.2E-02 | 4.3E-01 | 9.8E-01 | 9.6E-10 | 1.1E-02 | 2.0E-05 | 1.0E+00 | 9.5E-01 | 9.8E-01 |
| FOCAL_ADHESION | 7.4E-01 | 3.9E-01 | 5.2E-02 | 3.7E-02 | 3.1E-01 | 2.2E-02 | 2.8E-01 | 4.3E-01 | 9.8E-01 | 2.3E-06 | 4.3E-04 | 4.4E-06 | 1.0E+00 | 9.5E-01 | 9.8E-01 |
| GLYCOLYSIS_GLUCONEOGENESIS | 9.5E-01 | 1.9E-04 | 4.3E-04 | 4.0E-01 | 7.1E-05 | 2.0E-05 | 1.4E-02 | 4.3E-01 | 9.8E-01 | 2.4E-08 | 1.8E-02 | 2.7E-03 | 1.0E+00 | 9.5E-01 | 9.8E-01 |
| HEMATOPOIETIC_CELL_LINEAGE | 7.5E-01 | 1.0E-02 | 9.9E-02 | 2.4E-01 | 7.7E-02 | 1.1E-01 | 6.1E-02 | 8.8E-01 | 9.8E-01 | 7.8E-08 | 5.0E-03 | 1.8E-05 | 1.0E+00 | 9.5E-01 | 9.8E-01 |
| INTESTINAL_IMMUNE_NETWORK_FOR_IGA_PRODUCTION | 9.5E-01 | 1.2E-01 | 3.0E-03 | 3.0E-01 | 8.0E-01 | 7.1E-01 | 7.7E-02 | 1.2E-01 | 9.8E-01 | 7.5E-06 | 1.0E-01 | 5.8E-03 | 1.0E+00 | 9.5E-01 | 9.8E-01 |
| JAK_STAT_SIGNALING | 9.5E-01 | 6.8E-04 | 2.0E-02 | 4.8E-01 | 1.5E-01 | 3.9E-01 | 1.3E-01 | 7.3E-02 | 9.8E-01 | 2.7E-05 | 1.4E-02 | 4.4E-02 | 1.0E+00 | 9.5E-01 | 9.8E-01 |
| LYSOSOME | 7.5E-01 | 4.7E-03 | 8.6E-05 | 2.0E-03 | 3.4E-01 | 1.7E-06 | 2.2E-02 | 8.8E-01 | 6.8E-01 | 2.5E-12 | 7.7E-09 | 2.6E-10 | 1.0E+00 | 9.5E-01 | 9.8E-01 |
| MAPK_SIGNALING | 7.5E-01 | 5.1E-03 | 4.9E-02 | 2.4E-01 | 3.1E-01 | 7.2E-02 | 5.3E-02 | 1.6E-01 | 9.8E-01 | 7.3E-09 | 9.9E-01 | 1.8E-05 | 1.0E+00 | 9.5E-01 | 9.8E-01 |
| MISMATCH_REPAIR | 7.8E-01 | 4.3E-04 | 2.0E-04 | 4.0E-01 | 4.5E-02 | 1.9E-03 | 1.3E-01 | 3.7E-01 | 9.8E-01 | 4.1E-06 | 5.3E-01 | 1.5E-02 | 1.0E+00 | 9.5E-01 | 9.8E-01 |
| MTOR_SIGNALING | 8.7E-01 | 3.8E-02 | 5.8E-02 | 5.5E-01 | 3.0E-01 | 3.3E-01 | 1.4E-01 | 4.4E-01 | 9.8E-01 | 7.8E-03 | 1.4E-01 | 8.7E-01 | 1.0E+00 | 9.5E-01 | 9.8E-01 |
| NATURAL_KILLER_CELL_MEDIATED_CYTOTOXICITY | 7.5E-01 | 2.0E-01 | 3.9E-02 | 8.4E-02 | 3.5E-04 | 1.4E-02 | 6.4E-01 | 8.8E-01 | 9.8E-01 | 7.9E-01 | 1.5E-01 | 5.8E-03 | 1.0E+00 | 9.5E-01 | 9.8E-01 |
| NOD_LIKE_RECEPTOR_SIGNALING | 7.5E-01 | 7.7E-05 | 7.5E-03 | 3.1E-01 | 2.3E-01 | 9.5E-03 | 3.3E-02 | 8.8E-01 | 9.8E-01 | 6.4E-05 | 6.0E-01 | 1.7E-02 | 1.0E+00 | 9.5E-01 | 9.8E-01 |
| NUCLEOTIDE_EXCISION_REPAIR | 8.9E-01 | 3.0E-03 | 1.7E-03 | 4.9E-01 | 9.5E-03 | 7.0E-03 | 2.2E-01 | 1.6E-01 | 9.8E-01 | 1.5E-04 | 7.9E-01 | 4.4E-02 | 1.0E+00 | 9.5E-01 | 9.8E-01 |
| OXIDATIVE_PHOSPHORYLATION | 7.4E-01 | 8.7E-06 | 8.6E-05 | 5.5E-01 | 8.0E-02 | 5.0E-01 | 1.1E-04 | 7.3E-02 | 9.8E-01 | 5.2E-07 | 1.1E-02 | 5.2E-03 | 1.0E+00 | 9.5E-01 | 9.8E-01 |
| PHOSPHATIDYLINOSITOL_SIGNALING_SYSTEM | 6.3E-01 | 1.1E-03 | 1.1E-02 | 4.9E-01 | 4.5E-02 | 3.6E-01 | 3.3E-02 | 2.9E-01 | 9.8E-01 | 9.7E-06 | 1.5E-01 | 4.4E-02 | 1.0E+00 | 9.5E-01 | 9.8E-01 |
| PPAR_SIGNALING | 7.0E-01 | 3.2E-01 | 5.2E-01 | 8.7E-01 | 5.1E-01 | 5.5E-01 | 9.0E-01 | 8.8E-01 | 9.8E-01 | 1.4E-03 | 1.1E-02 | 1.4E-01 | 1.0E+00 | 9.5E-01 | 9.8E-01 |
| PROTEASOME | 2.3E-02 | 6.7E-05 | 1.7E-03 | 4.9E-01 | 9.5E-03 | 8.3E-01 | 5.3E-02 | 7.3E-02 | 9.8E-01 | 1.0E-06 | 7.3E-01 | 1.2E-03 | 1.0E+00 | 9.5E-01 | 9.8E-01 |
| PROTEIN_EXPORT | 8.0E-01 | 1.9E-04 | 1.5E-03 | 1.9E-01 | 4.5E-02 | 1.9E-02 | 2.9E-01 | 4.3E-01 | 9.8E-01 | 4.7E-03 | 9.0E-01 | 2.2E-02 | 1.0E+00 | 9.5E-01 | 9.8E-01 |
| REGULATION_OF_AUTOPHAGY | 8.9E-01 | 8.5E-01 | 3.6E-01 | 6.0E-01 | 3.1E-01 | 4.3E-01 | 7.3E-01 | 8.8E-01 | 9.8E-01 | 9.1E-01 | 8.8E-01 | 7.8E-01 | 1.0E+00 | 9.5E-01 | 9.8E-01 |
| RIG_I_LIKE_RECEPTOR_SIGNALING | 9.5E-01 | 1.8E-03 | 1.0E-01 | 8.7E-01 | 9.1E-01 | 3.1E-01 | 4.6E-01 | 4.3E-01 | 9.8E-01 | 1.6E-01 | 1.8E-02 | 8.4E-01 | 1.0E+00 | 9.5E-01 | 9.8E-01 |
| RNA_DEGRADATION | 8.7E-01 | 4.3E-04 | 1.8E-03 | 2.5E-01 | 2.0E-05 | 1.3E-05 | 5.3E-02 | 5.2E-01 | 9.8E-01 | 1.6E-04 | 2.0E-01 | 1.4E-02 | 1.0E+00 | 9.5E-01 | 9.8E-01 |
| RNA_POLYMERASE | 6.3E-01 | 9.4E-02 | 9.5E-01 | 3.0E-01 | 7.5E-01 | 8.5E-01 | 1.3E-01 | 5.4E-01 | 9.8E-01 | 3.5E-03 | 2.5E-03 | 8.1E-03 | 1.0E+00 | 9.5E-01 | 9.8E-01 |
| SPLICEOSOME | 6.3E-01 | 5.5E-02 | 5.2E-02 | 8.4E-02 | 2.3E-01 | 1.4E-01 | 2.9E-01 | 7.4E-01 | 9.8E-01 | 8.1E-01 | 3.5E-03 | 3.3E-01 | 1.0E+00 | 9.5E-01 | 9.8E-01 |
| T_CELL_RECEPTOR_SIGNALING | 8.9E-01 | 9.4E-04 | 6.3E-02 | 4.5E-01 | 2.6E-03 | 5.5E-03 | 1.4E-02 | 4.4E-01 | 9.8E-01 | 3.9E-03 | 5.3E-04 | 4.9E-01 | 1.0E+00 | 9.5E-01 | 9.8E-01 |
| TGF_BETA_SIGNALING | 7.3E-01 | 1.7E-01 | 5.0E-01 | 8.7E-01 | 3.1E-01 | 3.3E-01 | 2.0E-01 | 8.8E-01 | 9.8E-01 | 8.0E-02 | 9.9E-01 | 7.7E-01 | 1.0E+00 | 9.5E-01 | 9.8E-01 |
| TOLL_LIKE_RECEPTOR_SIGNALING | 7.0E-01 | 4.3E-05 | 3.0E-03 | 3.0E-01 | 6.0E-01 | 7.2E-02 | 6.1E-02 | 4.3E-01 | 9.8E-01 | 2.3E-06 | 8.5E-01 | 3.4E-02 | 1.0E+00 | 9.5E-01 | 9.8E-01 |
| UBIQUITIN_MEDIATED_PROTEOLYSIS | 8.9E-01 | 3.8E-02 | 2.0E-02 | 4.1E-01 | 3.5E-04 | 1.7E-06 | 4.9E-01 | 6.8E-01 | 9.8E-01 | 7.8E-02 | 5.0E-02 | 8.6E-03 | 1.0E+00 | 9.5E-01 | 9.8E-01 |
| VEGF_SIGNALING | 7.5E-01 | 2.6E-02 | 1.6E-01 | 3.0E-01 | 7.7E-02 | 1.9E-01 | 1.1E-01 | 8.8E-01 | 9.8E-01 | 1.9E-03 | 6.0E-01 | 1.1E-01 | 1.0E+00 | 9.5E-01 | 9.8E-01 |

1. Hochberg Y and Benjamini Y. More powerful procedures for multiple significance testing. Stat Med. 1990; 9:811-818.
