# Supplementary material for: Aging-dependent alterations in gene expression and a mitochondrial signature of responsiveness to human influenza vaccination
Source: Aging (Albany NY). 2015 Jan 14;7(1):38–51. doi: 10.18632/aging.100720 (PMC4356402; doi:10.18632/aging.100720)
Supplement: Supplementary file 7 [file aging-07-38-s007.pdf]

SUPPLEMENTARY TABLES

Please browse the full text version of this manuscript to see the links to Supplementary Tables:

Supplementary Table 1. Differential expression analysis revealing increased (1), decreased (-1) or unchanged (0) gene expression at days 2,7 and 28 post-vaccination in young, older and frail adults classified as vaccine responders (R+) and non-responders (R-). Refer to methods for details of differential expression analysis.

Supplementary Table 2. Differential expression analysis of pre-vaccination transcriptional profiles in young vs. older adults revealing genes up-regulated (q<0.05 and |Fold-Change|≥1.25) in young and older adults.

Supplementary Table 3A. Pathway activities obtained by QuSAGE (FDR < 0.05 for at least one of the condition) for the Blood Transcriptional Modules (BTMs) defined in [1]. QuSAGE was used to quantify the activity of each module by comparing pre- and post-vaccination transcriptional profiles. Positive activity represents up-regulation and negative activity represents down-regulation.

Supplementary Table 3B. False Discovery Rates (FDRs) associated with vaccination-induced activities (FDR < 0.05 for at least one of the condition) for the Blood Transcriptional Modules (BTMs) defined in [1]. QuSAGE was used to obtain P values for detecting the activity of each module by comparing pre- and post-vaccination transcriptional profiles. FDRs were obtained using the method of Benjamini and Hochberg [2].

Supplementary Table 4A. Pathway activities obtained by QuSAGE for immunological pathways from the KEGG database by comparing pre- and post-vaccination transcriptional profiles. Note that this table includes all pathways, while Figure 4 includes a subset of these pathways with FDR<0.005.

Supplementary Table 4B. False Discovery Rates (FDRs) associated with vaccination-induced pathway activities. QuSAGE was used to obtain P values for detecting the activity of immunological pathways from the KEGG database by comparing pre- and post-vaccination transcriptional profiles. FDRs were obtained using the method of Benjamini and Hochberg [1]. Note that this table includes all pathways, while Figure 4 includes a subset of these pathways with FDR<0.005.

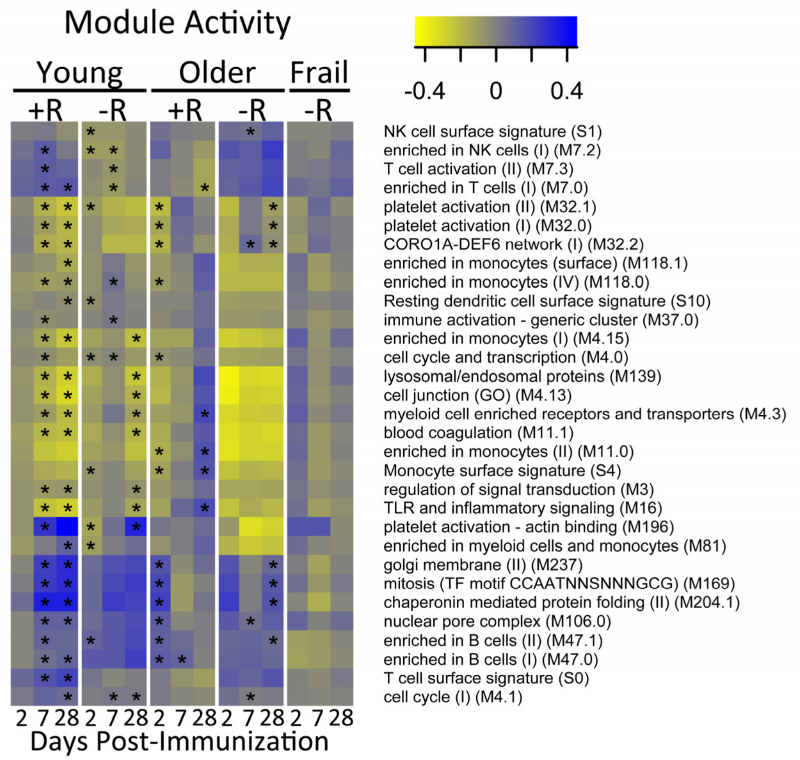

Supplementary Figure 1. QuSAGE [1] was used to screen Blood Transcription Modules (rows) [2] to identify modules with significantly (FDR<0.0001) altered activity for at least two conditions. Coloring represents down-regulated (yellow) to up-regulated (Blue) activity relative to the pre-vaccination time-point. +R indicates vaccine responder status, -R indicates non-responder status. Time points wirh FDR <0.05 are indicated with an asterisk.

1. Yaari G, Bolen CR, Thakar J and Kleinstein SH. Quantitative set analysis for gene expression: a method to quantify gene set differential expression including gene-gene correlations. Nucleic Acids Res. 2013; 41:e170.

2. Li S, Rouphael N, Duraisingham S, Romero-Steiner S, Presnell S, Davis C, Schmidt DS, Johnson SE, Milton A, Rajam G, Kasturi S, Carlone GM, Quinn C, et al. Molecular signatures of antibody responses derived from a systems biology study of five human vaccines. Nat Immunol. 2014; 15:195-204.
